# The function of Mak16 in ribosome biogenesis depends on its [4Fe-4S] cluster

**DOI:** 10.1073/pnas.2513844122

**Published:** 2025-11-13

**Authors:** Nadine Duppe, Lukas Knauer, Marc Hagebölling, Lena Langner, Martin Stümpfig, Volker Schünemann, Antonio J. Pierik, Daili J. Netz

**Affiliations:** ^a^Department of Chemistry, Rheinland-Pfälzische Technische Universität Kaiserslautern-Landau, Kaiserslautern 67663, Germany; ^b^Department of Physics, Rheinland-Pfälzische Technische Universität Kaiserslautern-Landau, Kaiserslautern 67663, Germany; ^c^Institut für Zytobiologie und Zytopathologie, Fachbereich Medizin und Zentrum für Synthetische Mikrobiologie Synmikro, Philipps-Universität Marburg, Marburg 35032, Germany

**Keywords:** iron–sulfur, metallocofactor, ribosome biogenesis

## Abstract

Mak16 and its interacting partner Rpf1 play a critical role at an early step in the maturation of the ribosomal 60S subunit of eukaryotes, as revealed by cryoelectron microscopy structures. While these studies suggested no metal participation or the presence of a Zn^2+^ ion in Mak16, we identify a previously unexplored iron–sulfur (Fe/S) cluster in yeast Mak16 through both in vivo and in vitro methods. We demonstrate a functional link between mitochondrial and cytosolic Fe/S protein biogenesis and ribosome assembly, highlighting an overlooked aspect of 60S ribosomal biogenesis. Characterization of human and yeast Mak16 revealed a redox-active [4Fe-4S]^2+/1+^ cluster with a midpoint potential below –500 mV. Oxidative stress destabilizes Mak16 and disrupts its interaction with Rpf1 in vivo, while in vitro H_2_O_2_ causes [3Fe-4S]^1+^ cluster formation. Our findings also reveal that upon binding to rRNA expansion segment 7 the redox properties of the nearby Fe/S cluster largely remain unchanged. However, disruption of Fe/S cluster coordination destabilized Mak16, impaired the Mak16–Rpf1 complex formation and decreased the 25S rRNA level. The critical role of Fe/S proteins in eukaryotic DNA replication and repair, mitoribosomal function, and maturation has now been extended to nuclear ribosomal assembly. Relying on a vulnerable cofactor comes at a cost, as cluster loss can severely disrupt essential cellular processes. The inherent biosynthetic complexity and instability of the Fe/S cluster of Mak16 allows it to function as sensor for redox imbalance, creating the possibility to regulate cellular homeostasis under stress.

## Significance

This study identifies an iron–sulfur (Fe/S) cluster in the 60S ribosomal assembly factor Mak16 as essential for maintaining complex stability with its interacting partner Rpf1, which is crucial for the maturation of 25S ribosomal RNA (rRNA) during nuclear ribosome biogenesis. The [4Fe-4S] cluster of Mak16 displays redox properties typical of low-potential centers and is highly labile in the presence of oxygen and oxidative stress-inducing agents. Together, these features suggest that the Fe/S cluster of Mak16 plays a structural and potentially regulatory role in ribosome assembly. Our findings uncover an unappreciated requirement for an Fe/S cofactor in a key process in eukaryotic cells and expand the importance of Fe/S protein biosynthesis.

Iron–sulfur (Fe/S) clusters are ancient and essential metal cofactors, found in more than 100 eukaryotic proteins which fulfill critical functions in hallmark processes such as photosynthesis, citric acid cycle, and respiration (1). In addition to their established roles in electron transport, enzymatic catalysis, and gene regulation, Fe/S-containing proteins are pivotal in maintaining genomic stability by participating in DNA replication and repair, where they are critical cofactors of DNA polymerases, primase, helicases, and glycosylases (2). Fe/S proteins also play critical roles in transcription [RNA polymerase III (3) and transcription factor IIH (4)]. In the cytoplasm of yeast and humans the Rli1 protein utilizes its two [4Fe-4S] clusters to functionally support key stages of ribosome recycling (5), ensuring seamless transitions between initiation and elongation (6). Structural proteins of the human 28S and 39S mitochondrial ribosomal subunits harbor two and one [2Fe-2S] clusters, respectively, which enhance stability and support translational fidelity (7, 8). Moreover, the assembly of the small mitoribosomal subunit requires the [4Fe-4S] protein METTL17 (9).

In yeast, the assembly of the 40S and 60S ribosomal subunits begins in the nucleolus with the transcription of 35S rRNA, which contains the 18S, 5.8S, and 25S rRNAs. This process is guided by more than 100 accessory ribosomal proteins, that coordinate spatiotemporal events in ribosome maturation (10–12). The complexity of this biological process is enhanced by the involvement of metal ions, necessary for RNA folding, chemical modification, stability, and precise assembly (13). Mg^2+^ ions, which bind both to ribonucleotides and proteins, are extensively distributed in ribosomes. In contrast, transition metals such as Zn^2+^ are only coordinated by proteins (14). In recent years, several cryoelectron microscopy (cryo-EM) structures depicting distinct ribosomal precursors of the small and large subunits at various stages have contributed to the understanding of ribosome maturation (15–18). At an early stage, a key cotranscriptional assembly checkpoint is established by the binding of the Noc1-Noc2 assembly factors to Rrp5 (19). This checkpoint occurs just before 35S rRNA cleavage within the internal transcribed spacer 1, leading to the separation of the small and large ribosomal subunits. This event triggers the recruitment of a module consisting of Mak16, its partner protein Rpf1, the β-propeller protein Nsa1, and the α-helical protein Rrp1, which spatially constrains domains I and II of the 25S rRNA precursor (19).

Mak16 captured our attention due to the coimmunoprecipitation of the human homologue (MAK16) with the MMS19, CIA2B, and CIAO1 proteins, which form the Cytosolic Iron–sulfur protein Assembly (CIA) targeting complex responsible for the final step of Fe/S cluster insertion into apo-proteins (20, 21). In its N-terminal domain (NTD), Mak16 features a highly conserved cysteine motif capable of binding a Zn^2+^ ion or an Fe/S cluster. At a first glance, the hypothesis that Mak16 could be an Fe/S protein appeared incorrect, as in most cryo-EM structures Mak16 was either devoid of a metal ion, or Zn^2+^ binding was proposed (16–18). The resolution in cryo-EM structures poses challenges for precise metal ion positioning and identification due to the near-identical coordination environment for both Zn^2+^ and an Fe/S cluster. In this study, we performed a comprehensive analysis of the biophysical properties of recombinant yeast and human Mak16. We further interrogated in vivo Fe/S cluster assembly on Mak16 and explored the cellular consequences of defective assembly for Mak16 function in yeast. Our findings clarify the central question of metal occupancy in Mak16 and represent an important step toward understanding of the physiological significance of the Fe/S cluster of Mak16 in the context of 60S ribosome maturation.

## Results

### Mak16 from Different Eukaryotes Binds a Bona Fide Fe/S Cluster.

**Fig. 1.**
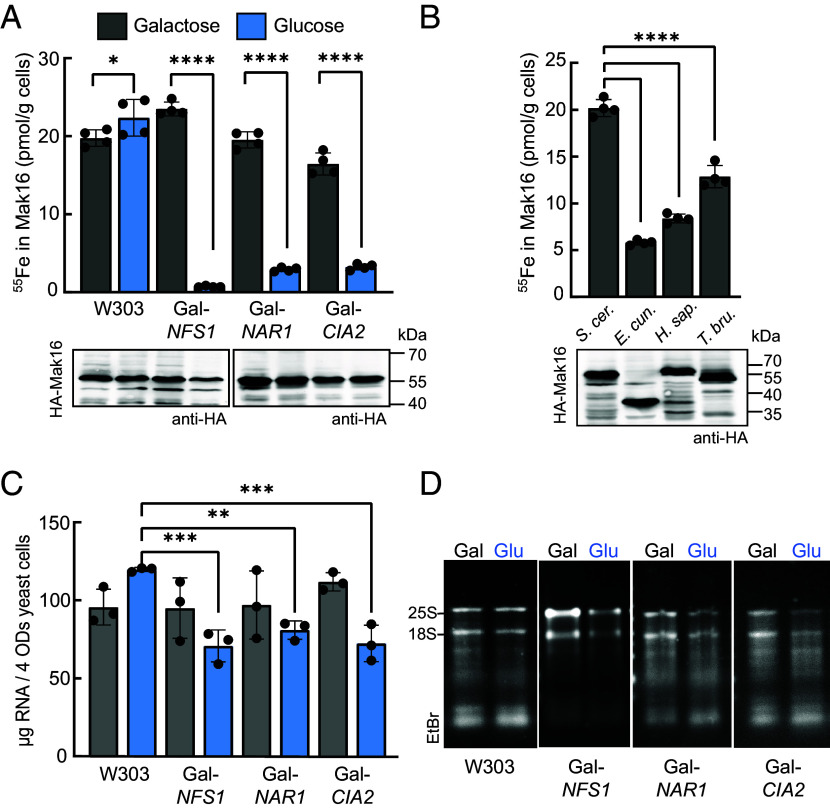
Assembly of Fe/S clusters on Mak16 and efficient rRNA synthesis relies on the ISC and CIA machinery pathways. (*A*) Incorporation of ^55^Fe in Mak16 in yeast strains expressing or depleted for ISC- or CIA-factors. The 416*MET25*-HA-Mak16 plasmid was transformed into W303, Gal-*NFS1*, Gal-*NAR1,* and Gal-*CIA2* yeast cells, grown in iron-poor SC medium supplemented with galactose or glucose and ^55^FeCl_3_. HA-Mak16 was immunoprecipitated with anti-HA beads from cell extracts. Data are mean value ± SEM (*n* = 4). Significance of differences between groups, as indicated by crossbars, were determined using multiple comparisons one-way ANOVA with Šidák correction (*P < 0.05, ****P < 0.0001). (*B*) ^55^Fe incorporation in Mak16 homologues expressed in W303 yeast cells in glucose containing SC medium as in (*A*). Abbreviations: *S. cer.*, *S. cerevisiae; H. sap.*, *Homo sapiens*; *E. cun.*, *Encephalitozoon cuniculi*; *T. bru.*, *Trypanosoma brucei*. ****P < 0.0001. (*C*) Total RNA isolated from W303 and yeast strains expressing or depleted for ISC- or CIA-factors. Yeast cells were cultured for 24 h in SC medium containing either galactose or glucose. Total RNA was then extracted from cells after two doubling times. *n* = 3, ***P < 0.001, **P < 0.01. (*D*) From the same samples used in (*C*) the RNA was separated by agarose gel electrophoresis and detected by GelRed staining.

The most effective approach to identify iron or Fe/S association with a eukaryotic protein is to analyze the full-length protein in its native organism (22). To this end, N-terminally HA-tagged yeast Mak16 was expressed from a single-copy plasmid under control of the *MET25* promoter in wild-type (W303) yeast cells. Radioactive ^55^Fe was incorporated into cells, and its specific association with Mak16 was demonstrated through anti-HA immunoprecipitation. The analysis detected substantial ^55^Fe binding to Mak16 at a level of 19 to 25 pmol/g of cells (Fig. 1*A*). Due to the approximately 20% efficiency of cell lysis, losses of 3xHA-Mak16 and its [4Fe–4S] cluster during immunoprecipitation, and residual Fe present even in Fe-free medium, the measured ^55^Fe content cannot be directly converted into cluster occupancy per molecule. Nevertheless, the in vivo ^55^Fe levels of Mak16 are consistent with those observed for other well-characterized yeast Fe/S proteins such as Pol3, Leu1, Rli1, Cfd1, Nbp35, Ntg2, and Nar1 [3 to 35 pmol/g (22, 23)] when expressed as full-length proteins in their native environments. Furthermore, as previously demonstrated (23), inadvertent ^55^Fe incorporation into cysteine-rich proteins, including those with multiple zinc fingers, does not take place under these conditions.

To determine whether the ^55^Fe originated from mononuclear, dinuclear, or Fe/S iron ions, we examined the dependence of ^55^Fe-association with Mak16 on key Fe/S protein biogenesis factors. These included Nfs1, required for the assembly of all Fe/S proteins, and the CIA components Nar1 and Cia2, involved in cytosolic and nuclear Fe/S protein maturation (24). For this purpose, galactose-regulatable strains were grown under permissive and repressive conditions (in galactose and glucose containing media, respectively, Fig. 1*A* and *SI Appendix*, Fig. S1). Upon efficient depletion of Nfs1, Nar1, or Cia2 proteins to undetectable levels on western blot (*SI Appendix*, Fig. S1 *C–E*), sufficient to cause biosynthetic phenotypes (22, 25, 26), ^55^Fe incorporation into Mak16 was significantly impaired. This defect is consistent with the interconnected roles of the mitochondrial ISC and CIA pathways, establishing Mak16 as a bona fide Fe/S protein in vivo.

We then questioned whether Mak16 from phylogenetically distant homologues (*SI Appendix*, Fig. S2*A*), including human Mak16, also bind an Fe/S cluster upon expression in yeast cells. Significantly lower quantities of ^55^Fe compared to yeast Mak16 were found in association with Mak16 homologs from humans, the microsporidial parasite *Encephalitozoon cuniculi*, and the kinetoplastic parasite *Trypanosoma brucei* (Fig. 1*B*, 6 to 13 pmol ^55^Fe/g). These differences do not arise from variations in expression levels (Fig. 1 *B*, *Bottom* and *SI Appendix*, Fig. S2*B*), but may result from a diminished stability of the Fe/S cluster of Mak16 from these organisms, or the more effective Fe/S incorporation into yeast Mak16 by its own machinery in comparison with Fe/S incorporation into Mak16 from other organisms. Interestingly, Mak16 proteins (39 to 42 kDa) exhibit unusually low electrophoretic mobility on sodium dodecyl sulfate polyacrylamide gel electrophoresis (SDS-PAGE), probably due their acidic nature (pI ~ 4.9), except for Mak16 from *E. cuniculi* (33 kDa, pI = 8.7), which lacks the acidic C-terminal domain (Fig. 1*B*). To test whether these homologues, including human Mak16 (49% amino acid identity), could functionally substitute for yeast Mak16, a Gal-*MAK16* yeast strain was generated. In this galactose-regulatable strain, the endogenous *MAK16* promoter was replaced with the *GALL* promoter. Under permissive conditions (galactose-containing medium), cells transformed with plasmids expressing Mak16 homologues from different organisms, driven by either the native *MAK16* or the *MET25* promoter, showed no obvious growth differences (*SI Appendix*, Fig. S2*C*). Strikingly, upon depletion of endogenous yeast Mak16 in glucose-containing medium, only the ectopically expressed yeast Mak16 was able to rescue growth. None of the three Fe/S cluster-binding homologues, including human Mak16, restored viability, underscoring the species-specific functional integration of Mak16 in ribosome biogenesis (*SI Appendix*, Fig. S2*C*). Since yeast Mak16 functions in concert with Rpf1 and other ribosomal maturation factors (16), the inability of Mak16 homologues to rescue the loss of endogenous Mak16 may stem from a failure to engage these species-specific interaction partners. In summary, while the presence of an Fe/S cluster is a conserved feature of Mak16 proteins across phylogenetically diverse organisms, their functional integration into ribosome biogenesis appears to depend on precise molecular interactions with coevolved assembly factors.

To further assess whether impaired Fe/S cluster assembly into yeast Mak16 affects rRNA synthesis, total RNA was isolated from exponentially growing yeast cells. RNA levels were compared in glucose-grown cells following depletion of selected ISC and CIA components, specifically Nfs1, Cia2, and Nar1 (Fig. 1*C*). Both Nfs1 and Cia2 depletion resulted in strong decrease of total RNA levels, while the effect of Nar1 depletion, possibly due to a slightly slower onset, was less pronounced. Under permissive conditions (galactose), where Fe/S biogenesis remains active, total RNA was largely composed of rRNA [~80% (27)], with prominent 25S and 18S bands visible on denaturing gels (Fig. 1*D* and *SI Appendix*, Fig. S3). In glucose medium upon depletion of these Fe/S assembly factors, both 25S and 18S rRNA signals were diminished, with the 25S rRNA most strongly affected, despite equal total RNA loading. These results indicate that disruption of Fe/S cluster biogenesis impairs Mak16 function in ribosome biogenesis and reduces rRNA synthesis efficiency.

### Human and Yeast Mak16 Proteins Contain a [4Fe-4S]^2+/1+^ Cluster.

**Fig. 2.**
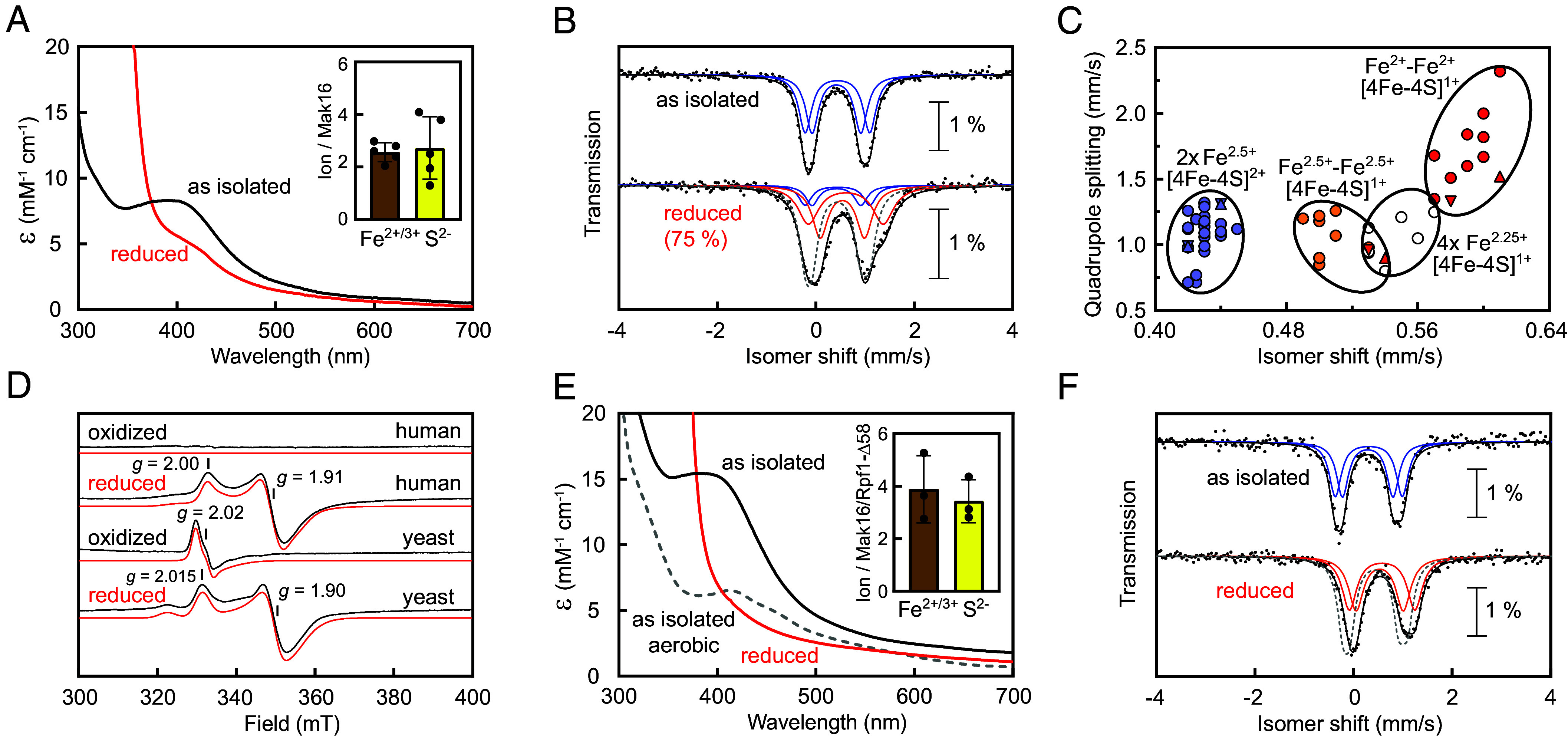
Yeast and human Mak16 contain a [4Fe-4S]^2+^ cluster. (*A*) UV-Vis spectrum of anaerobically purified human Mak16 (0.037 mM, black line, as isolated) in 50 mM sodium phosphate, 300 mM NaCl, pH 8.0, and after incubation with 4 mM sodium dithionite (red line, reduced). Inset: Nonheme (Fe^2+/3+^) and acid labile sulfide (S^2−^) contents (mean ± SEM, *n* = 5). (*B*) Mössbauer spectra at 77 K in the absence of an applied field of as isolated and partially reduced human Mak16 as in (*A*) but at ~0.4 mM concentration. Fits shown as lines: blue, [4Fe-4S]^2+^; orange/red, [4Fe-4S]^1+^; black, sum of components; dotted gray, sum of components for as isolated for comparison. Parameters in *SI Appendix*, Table S1. (*C*) Compilation of Mössbauer parameters for all-cysteine coordinated [4Fe-4S]^2+^ (blue) and [4Fe-4S]^1+^ clusters (orange, Fe^2.5+^ pair; red Fe^2+^ pair; open for systems simulated with a single quadrupole doublet); Human Mak16, triangles; yeast Mak16, inverted triangles. Values and references in *SI Appendix*, Tables S2 and S4. (*D*) EPR spectra of 0.5 mM potassium ferricyanide oxidized or 4 mM sodium dithionite treated human Mak16 (0.128 mM) or yeast Mak16/Rpf1-Δ58 complex (0.074 mM). EPR conditions: 9.353 GHz, 10 K, microwave power 0.21 mW. Parameters for simulations (red lines) in *SI Appendix*, Table S3. (*E*) UV-Vis spectrum of anaerobically purified as isolated (black line), dithionite-reduced (red line), and aerobically purified yeast (dashed line) Mak16/Rpf1-Δ58 complex (0.011 mM), as in (*A*). Fe^2+/3+^ and S^2-^ contents for anaerobically purified complex (mean ± SEM, *n* = 3). (*F*) Mössbauer spectroscopy of anaerobically purified as isolated and reduced yeast Mak16/Rpf1-Δ58 complex (~0.6 mM) as in (*B*).

To investigate the Fe/S cluster type and its biophysical properties, we expressed N-terminally hexahistidine tagged human and yeast Mak16 in *Escherichia coli*. Human Mak16 could be affinity purified under anaerobic conditions in absence of its interaction partners (*SI Appendix*, Fig. S4*A*). Ultraviolet-Visible (UV-Vis) spectroscopy of the beige protein showed a well-defined shoulder with a maximum at 400 nm (Fig. 2*A*), which was partially bleached upon dithionite reduction. The absence of characteristic UV-Vis absorption peaks at 330, 450, and 550 nm strongly suggests that human Mak16 does not harbor a [2Fe-2S] cluster, as reflected by its broad, featureless spectrum. The iron and acid-labile sulfide contents (2.6 ± 0.4 and 2.7 ± 1.2, respectively), together with an experimental extinction coefficient at 400 nm (ε_400 nm_ ~ 8.5 mM^−1^cm^−1^), indicate that purified human Mak16 contains either a nearly fully occupied [3Fe-4S]^1+^ (ε_400 nm_ ~ 9.8 mM^−1^cm^−1^ per 3Fe), or a partially occupied [4Fe-4S]^2+^ cluster (ε_400 nm_ ~ 13 mM^−1^cm^−1^ per 4Fe). The Mössbauer spectrum of human Mak16 isolated from ^57^Fe-grown *E. coli* cells could be simulated with two equal intensity quadrupole doublets with almost identical isomer shifts (δ_1_ = 0.42 and δ_2_ = 0.44 mm/s) and slightly differing quadrupole splitting (ΔE_Q1_ = 0.99 and ΔE_Q2_ = 1.31 mm/s, Fig. 2*B* and *SI Appendix*, Table S1). These Mössbauer parameters differ from those of [2Fe-2S]^2+^ and [3Fe-4S]^1+/0^ clusters (28), but closely match the valence delocalized Fe^2+^-Fe^3+^ pairs (2× Fe^2.5+^) of all cysteine-coordinated protein-bound [4Fe-4S]^2+^ clusters (Fig. 2*C* and *SI Appendix*, Table S2). The lack of a signal at *g* = 2.02 in the electron paramagnetic resonance (EPR) spectrum of ferricyanide oxidized human Mak16 corroborated the absence of [3Fe-4S]^0/1+^. In agreement with the observed bleaching of the UV-Vis spectrum, dithionite reduction generated a prominent axial EPR signal with *g*_z_ = 2.00 and *g*_yx_ = 1.91 for the [4Fe-4S]^1+^ cluster (Fig. 2*D*, EPR simulation parameters *SI Appendix*, Table S3). Dithionite treatment reduced approximately 75% of the [4Fe-4S] cluster, as evidenced by changes in the Mössbauer spectrum. Specifically, the isomer shifts of both quadrupole doublets increased upon reduction, reflecting changes in the electronic environment of the iron centers. Notably, the diferrous pair exhibited a larger increase in isomer shift compared to the other doublet, which remained intermediate between the values for mixed-valence Fe^2.5+^ and diferrous Fe^2+^ pairs found in biological [4Fe-4S]^1+^ clusters (Fig. 2*C* and *SI Appendix*, Table S4). Taken together, these findings show that human Mak16 contains a single cluster, with stable [4Fe-4S]^1+^ and [4Fe-4S]^2+^ oxidation states.

In contrast to human Mak16, yeast Mak16 proved difficult to purify to homogeneity (*SI Appendix*, Fig. S4*B*) and exhibited an unstable Fe/S cluster, with less than one iron per monomer *SI Appendix*, Fig. S4*C*). However, coexpression with its Brix-domain partner protein, yeast Rpf1, significantly enhanced the protein homogeneity (*SI Appendix*, Fig. S4*D*). Western blot analysis and Coomassie-stained SDS-PAGE showed a slow-migration His-Mak16 band (~50 kDa, despite a calculated mass of 37.3 kDa) alongside a distinct ~35 kDa band corresponding to Rpf1 (*SI Appendix*, Fig. S4*E*). Truncating the two N-terminal helices of Rpf1 (Rpf1-Δ58, *SI Appendix*, Fig. S5) further improved the homogeneity of the complex, resulting in a content of 3.9 ± 1.3 iron and 3.4 ± 0.8 sulfide per heterodimer. UV-visible (ε_400nm_ ~15 mM^−1^cm^−1^), and Mössbauer spectroscopy equally demonstrated the presence of a [4Fe-4S]^2+^ cluster in the yeast Mak16/Rpf1-Δ58 complex (Fig. 2 *E* and *F*). Notably, the Fe/S cluster was highly oxygen-labile: aerobic purification led to a ~50% loss of its chromophore (Fig. 2*E*), underscoring its instability in the presence of oxygen. Though a weak [3Fe-4S]^1+^ EPR signal (~5%) at *g* = 2.02 emerged upon oxidation (Fig. 2*D*), the low temperature, high field Mössbauer spectra demonstrated the diamagnetism of the cluster in its as isolated state (*SI Appendix*, Fig. S6*A*). We interpret the low content of a [3Fe-4S]^1+^ cluster as a product of [4Fe-4S] cluster degradation. Similar to human Mak16, dithionite treatment resulted in an axial EPR signal with *g*_z_ = 2.015 and *g*_yx_ = 1.90 (Fig. 2*D*, EPR simulation parameters *SI Appendix*, Table S3). Although a minor signal of a *S* = 3/2 species was detected in highly concentrated dithionite reduced Mak16/Rpf1-Δ58 (*SI Appendix*, Fig. S7), double integration of the simulated spectrum indicated that it accounted for <2% of the intensity relative to the double integral of the *g* = 2 region. Spin quantification against Cu^2+^ showed that the *S* = 1/2 EPR signal closely matched the protein concentration (within 5% accuracy). Our findings demonstrate that the [4Fe-4S]^1+^ cluster is almost exclusively in the *S* = 1/2 spin state. The Mössbauer spectrum at 77 K was best simulated by two quadrupole doublets with δ_1_ = 0.53 mm/s, ΔE_Q,1_ = 0.96 mm/s and δ_2_ = 0.58 mm/s, ΔE_Q,2_ = 1.33 mm/s, assuming complete reduction to [4Fe-4S]^1+^. Although magnetic splitting blurred the Mössbauer spectrum at 4 K (*SI Appendix*, Fig. S6*B*), the absence of a central doublet from [4Fe-4S]^2+^ confirmed the extent of cluster reduction. In summary, heterologously expressed yeast and human Mak16 proteins contain an oxygen-labile [4Fe-4S]^2+/1+^ cluster.

### Disruption of Fe/S Cluster Coordination in Mak16 Strongly Decreases rRNA Levels.

**Fig. 3.**
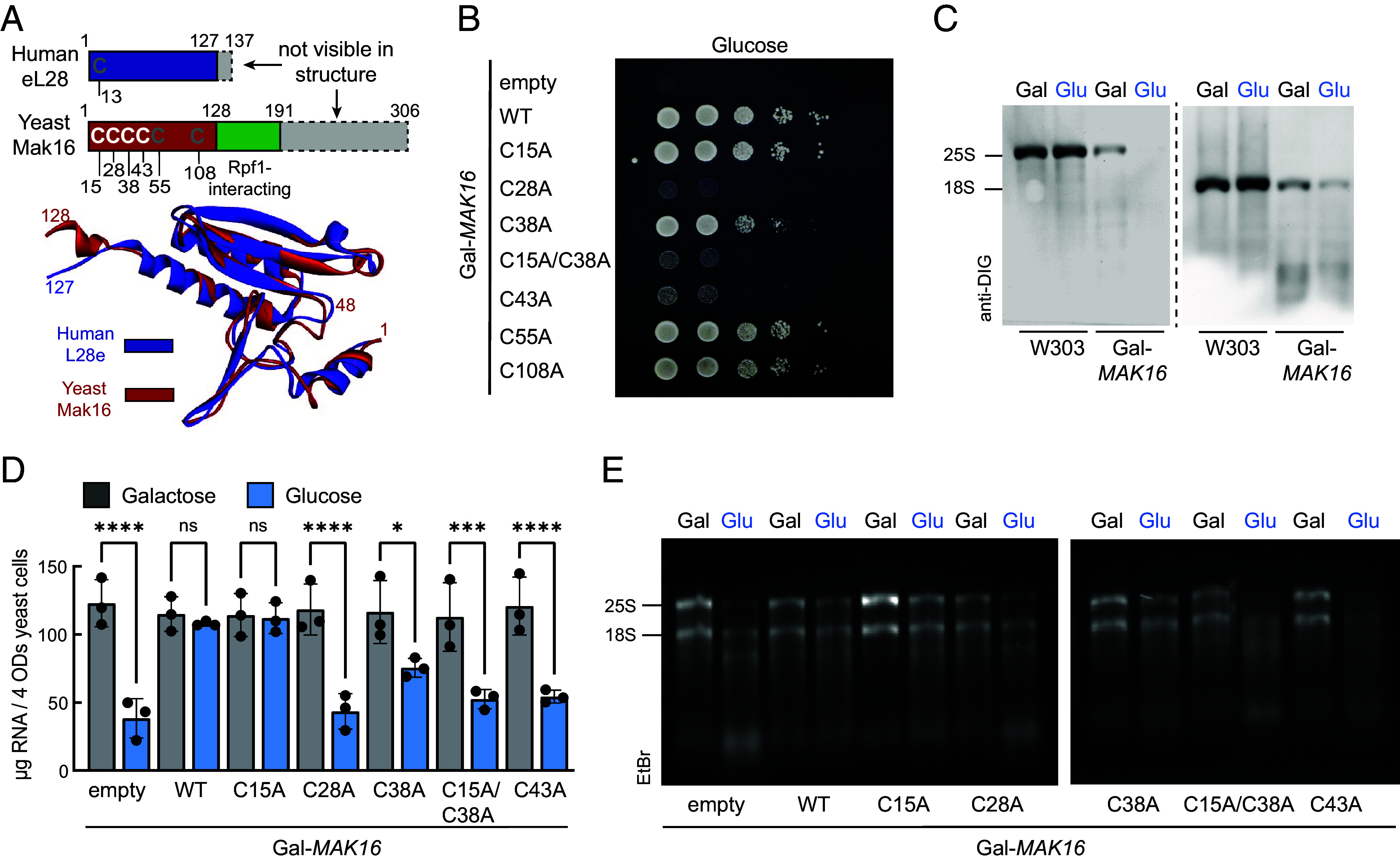
Disruption of [4Fe-4S] coordination in Mak16 leads to impaired cell growth and rRNA synthesis. (*A*, *Top*) conserved cysteine residues and domain structure of Mak16 in comparison with human L28e. (*Bottom*) Structural alignment of yeast Mak16 (PDB 6EM1) with the human ribosomal protein L28e (PDB 8G5Y). (*B*) Gal-*MAK16* yeast cells, transformed with 416 plasmids under control of the *MAK16* promoter lacking an insert (empty), or encoding wild-type (WT) or cysteine variants of Mak16, were cultured for 16 h in SC medium with glucose. OD-normalized 10-fold serial dilutions were spotted on agar plates of the same medium and photographed after 48 h at 30 °C. (*C*) Northern blots of total RNA isolated from wild-type (W303) and Gal-*MAK16* yeast cells cultivated for 40 h in SC medium with galactose (Gal) or glucose (Glu). rRNA was visualized with digoxigenin-ddUTP labeled 25S (*Left*) or 18S (*Right*) probes and anti-digoxigenin-peroxidase conjugate (*SI Appendix*, Fig. S10). (*D*) Quantification of total RNA isolated from cells grown for 24 h in galactose or glucose containing SC medium, followed by ~6 h of growth to the logarithmic phase. Data are mean value ± SEM (*n* = 3). Significance of differences between groups, as indicated by crossbars, were determined using multiple comparisons one-way ANOVA with Šidák correction (ns, not significant, *P < 0.05, ***P < 0.001, ****P < 0.0001). (*E*) Total RNA from samples in (*D*) was separated by agarose gel electrophoresis. rRNA bands were visualized with ethidium bromide (*SI Appendix*, Fig. S11).

Yeast Mak16 is a 306-amino acid protein composed of three domains. The N-terminal region (residues 1 to 128) adopts a knot-like fold that is structurally homologous to ribosomal protein L28e (Fig. 3*A*) and contains four invariant cysteine residues within residues 1 to 48, forming a CX_11-13_CX_9_CX_4_CP consensus motif (*SI Appendix*, Fig. S2*A*), which is consistent with the potential coordination of an Fe/S cluster. The central domain (residues 129 to 191) forms two α-helices that mediate interaction with Rpf1, while the C-terminal region is characterized by alternating stretches of basic and acidic residues. Structural predictions from AlphaFold2 show high agreement with cryo-EM data for the N-terminal and central domains. In contrast, the C-terminal region is predicted to be largely disordered and is absent from all cryo-EM reconstructions (*SI Appendix*, Fig. S8), likely due to its intrinsic flexibility and the limited capacity of cryo-EM to resolve dynamic or unstructured segments. This flexible domain is absent in Mak16 homologues from *Trypanosomatidae* and *Microsporidia*, suggesting that its role in ribosome biogenesis may vary across species.

To analyze the functional importance of the cysteine residues potentially involved in Fe/S cluster coordination, we assessed their role in vivo using the Gal-*MAK16* yeast strain. This strain was transformed with either an empty plasmid, plasmid encoding wild-type Mak16, or cysteine-to-alanine variants expressed under the control of the native *MAK16* promoter. Under permissive conditions (galactose) no obvious growth phenotype was observed *(**SI Appendix*, Fig. S9). However, under repressive conditions (glucose) substitutions of Cys28 or Cys43 by alanine resulted in a severe growth defect comparable to empty plasmid control (Fig. 3*B*). Replacement of Cys38 produced an intermediate phenotype, which was further exacerbated by an additional Cys15 substitution (C15A/C38A). In contrast, mutation of the nonconserved Cys55 or Cys108 had no detectable effect. The findings indicate that cysteine residues 15, 28, 38, and 43 within the CX_11-13_CX_9_CX_4_CP consensus motif are essential for coordinating the [4Fe-4S]^2+^ cluster. Together, these data underscore the critical role of a conserved cysteine-based motif in stabilizing the Fe/S cluster and enabling Mak16 function.

Given that rRNAs constitute the majority of cellular RNA, we examined whether the phenotypic severity of Mak16 cysteine variants (Fig. 3*B*) correlates with total RNA levels. Yeast cells depleted of endogenous Mak16 were harvested during early exponential growth, and total RNA was extracted. In parallel, control RNA was isolated from cells grown in galactose. Northern blotting revealed reduced 25S and, to a lesser extent, 18S rRNA levels upon depletion of wild-type Mak16 (Fig. 3*C* and *SI Appendix,* Fig. S10). Quantitative analysis showed a substantial decrease in total RNA in cells transformed with the empty plasmid (65% reduction), as well as the C28A, C38A, C15A/C38A, and C43A variants (30 to 60% reduction) (Fig. 3*D*). Contrarily, the C15A variant retained wild-type RNA levels. The RNA content paralleled the degree of growth complementation across the different Mak16 variants (Fig. 3 *B* and *D*). Agarose gel electrophoresis showed that both 25S and 18S rRNAs were affected, with 25S rRNA being the most severely impacted (Fig. 3*E*). In conjunction with the ISC/CIA depletion data (Fig. 1 *A*, *C*, and *D*), these findings identify the Fe/S cluster of Mak16 as a critical determinant for proper ribosome biogenesis and thus maintaining cellular rRNA levels.

### Mak16/Rpf1 Maintains a Low Redox Potential Independent of Extension Segment 7 (ES7) Binding.

**Fig. 4.**
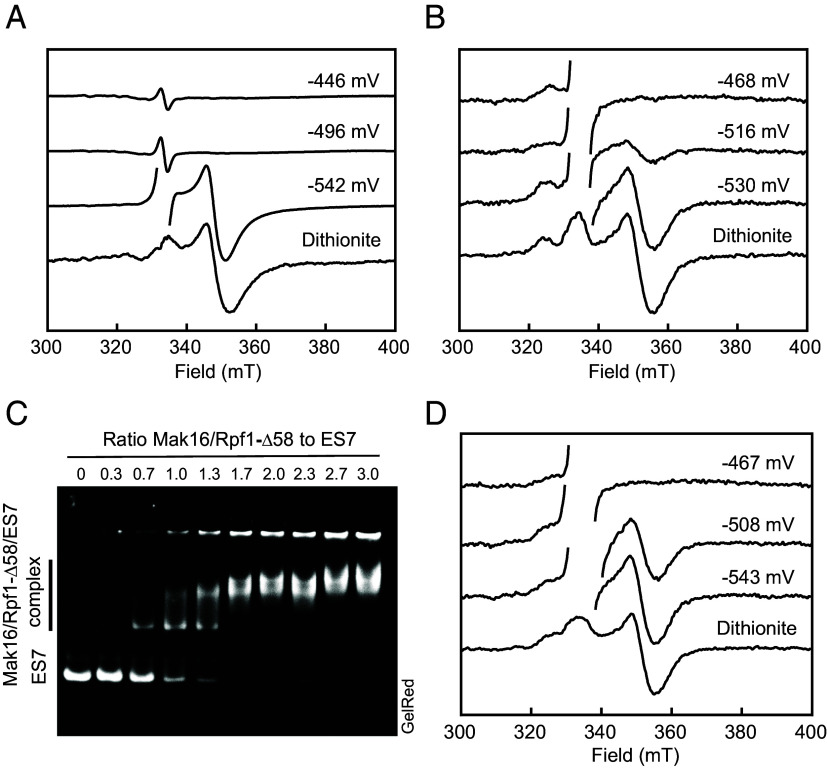
Redox properties of Mak16. (*A*) EPR-mediated redox titration. Human Mak16 (16 µM) in pyrophosphate buffer pH 9.0 was mixed with redox mediators, and the reduction potential was adjusted with sodium dithionite. EPR spectra for samples taken at indicated potentials are shown. EPR conditions: 9.356 GHz, 10 K, microwave power 0.21 mW. (*B*) EPR-mediated redox titration of yeast Mak16/Rpf1-Δ58 complex (9 µM) as in (*A*). (*C*) Binding of the Mak16/Rpf1 complex to ES7. Purified Mak16/Rpf1-Δ58 was titrated with a fixed amount of ES7 (0.8 µg) in binding buffer. The protein/RNA complex was separated by 5% nondenaturing acrylamide gel electrophoresis, visualized with GelRed and recorded with a gel documentation system. The same gel was further stained with Coomassie dye (*SI Appendix*, Fig. S12). (*D*) Effect of ES7 binding on the redox potential of Mak16/Rpf1 complex. Purified Mak16/Rpf1-Δ58 complex and ES7 (7.3 µM each) were incubated in binding buffer, and subjected to a redox titration as in (*A*).

Accurate determination of the redox potential of the [4Fe-4S]^2+/1+^ cluster in both human Mak16 (Fig. 4*A*) and the yeast Mak16/Rpf1-Δ58 complex (Fig. 4*B*) proved challenging. First, the low redox potential of the cluster did prevent full reduction using viologen redox mediators, in contrast to near-complete reduction achieved after a 3-min incubation with sodium dithionite. Second, the chemical lability of the cluster limited the feasibility of prolonged dye-mediated titrations across multiple samples. Nevertheless, EPR spectroscopy unequivocally demonstrates that both human and yeast Mak16 proteins harbor clusters with similarly low redox potentials, estimated to have midpoint potentials near –520 mV vs. the normal hydrogen electrode (NHE). To explore whether rRNA binding modulates the redox properties of Mak16, we examined the interaction between the recombinant yeast Mak16/Rpf1-Δ58 complex and the 25S rRNA ES7, which is associated with Mak16/Rpf1 in cryo-EM structures (16). Electrophoretic mobility shift assays (EMSA) demonstrated the formation of a defined complex upon stoichiometric mixing with in vitro transcribed ES7 (Fig. 4*C*). This interaction resulted in only a slight shift in the midpoint potential from –520 to approximately –500 mV vs. NHE (Fig. 4*D*), indicating that ES7 binding has minimal impact on the redox characteristics of the [4Fe-4S]^2+/1+^ cluster. Since in the physiology of human and yeast cells there are no redox processes taking place at such low redox potentials, we propose that Mak16 operates physiologically with its cluster in the [4Fe-4S]^2+^ state.

### Synthetic Lethality Between CIA Machinery Depletion and Expression of the Mak16 C38A Variant.

**Fig. 5.**
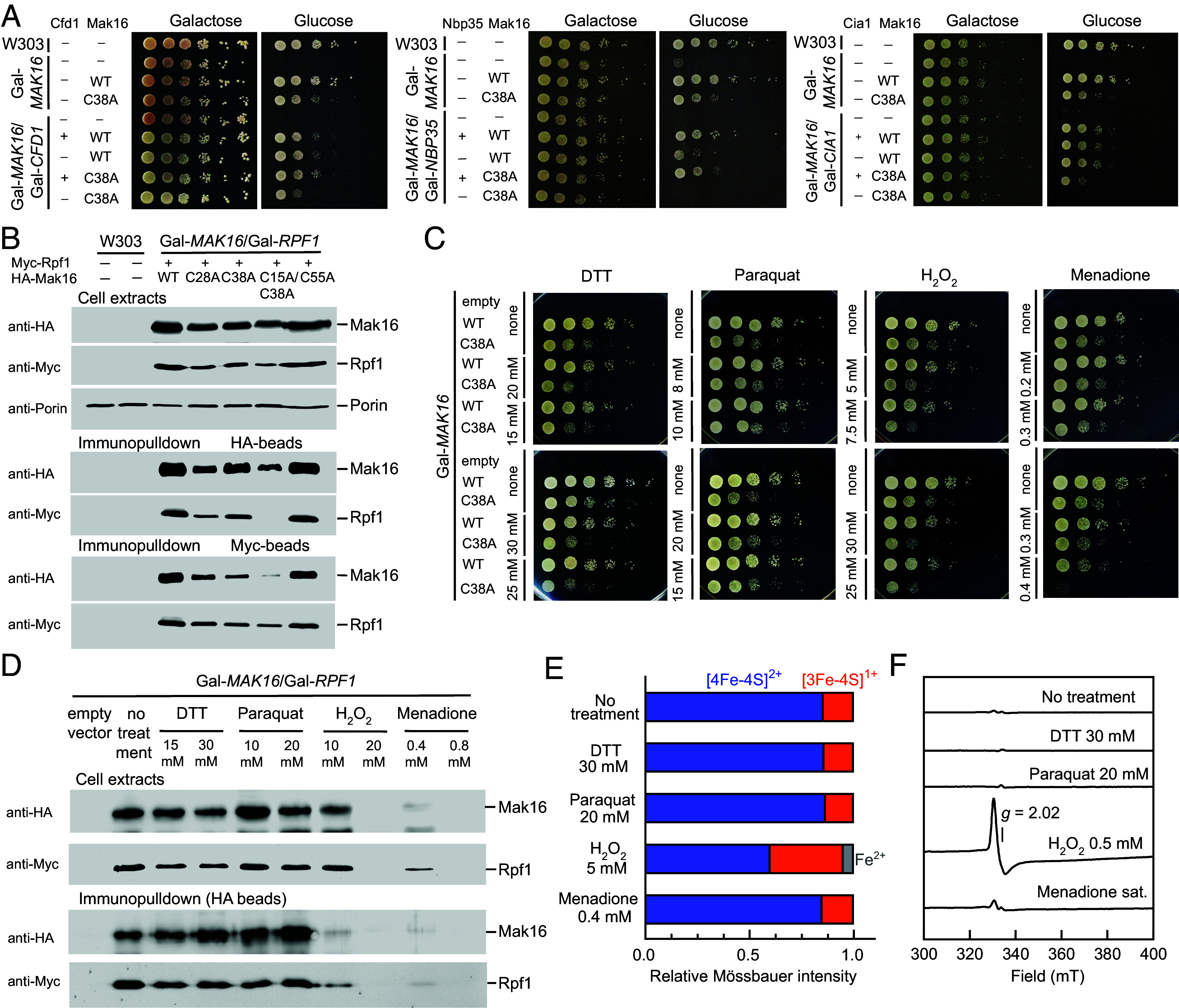
Effect of redox stressors on wild type Mak16, its C38A variant and Rpf1 binding. (*A*) Synthetic lethality between CIA machinery depletion and the Mak16 C38A variant. Indicated yeast strains were transformed with an empty, WT Mak16, or C38A Mak16 variant encoding plasmid, along with an empty plasmid or plasmid encoding the indicated CIA factor. After 16 h of growth on galactose or glucose, 10-fold serial dilutions were spotted on SC agar galactose or glucose plates and photographed after 48 h at 30 °C. (*B*) Disruption of Fe/S coordination in Mak16 impairs its interaction with Rpf1. W303 without plasmids or the Gal-*MAK16*/Gal-*RPF1* strain transformed with plasmids encoding WT or cysteine variants of HA-Mak16 and Myc-Rpf1 were grown for 40 h on glucose. Immunoprecipitates of cell extracts with either HA or Myc beads were subjected to western blot analysis with indicated antibodies (*SI Appendix*, Fig. S13). (*C*) Conditional lethality caused by expression of the Mak16 C38A variant and treatment with redox stressors. Gal-*MAK16* cells transformed with an empty plasmid, or a plasmid encoding WT, or the C38A Mak16 variant were grown on SC glucose for 16 h, then treated 2 h with indicated redox stressors, and 10-fold serial dilutions were spotted on SC glucose agar plates. After 48 h of growth at 30 °C plates were photographed. (*D*) Disruption of Fe/S of WT Mak16 by stressors in vivo impairs its stability and interaction with Rpf1. Gal-*MAK16*/Gal-*RPF1* cells transformed with empty plasmids or plasmids encoding WT HA-Mak16 and Myc-Rpf1 were grown and processed as in (*B*). Full blots in *SI Appendix,* Fig. S14. (*E*) Relative intensities of the quadrupole doublets at 77 K in the Mössbauer spectra (*SI Appendix*, Fig. S15) of purified ^57^Fe-enriched Mak16/Rpf1-Δ58 after treatment with indicated compounds (30 min., 23 °C). The sample used had a higher [3Fe-4S]^1+^ content than that in Fig. 2*F*. (*F*) EPR spectra of purified Mak16/Rpf1-Δ58 frozen after treatment with indicated compounds (30 min, 23 °C). EPR conditions: 9.352 GHz, 10 K, microwave power 0.21 mW.

To explore the functional interplay between defective Fe/S cluster coordination in Mak16 and impaired CIA machinery, we explored synthetic lethality effects on cell growth. For this purpose, yeast strains of which both *MAK16* and individual CIA genes were under control of the *GAL* promoter were constructed. Our focus was on the early-acting CIA factors Cfd1, Nbp35, and Cia1, whose depletion is known to produce relatively mild growth defects (29, 30). To probe additive or synergistic effects, we used the Mak16 C38A variant, which exhibits an intermediate growth phenotype in drop tests (Fig. 3*B*). In the Gal-*MAK16* background, the C38A mutation alone led to a growth reduction of approximately two serial dilutions (Fig. 5*A*). However, concurrent depletion of any of the three CIA factors markedly worsened this phenotype, resulting in growth comparable to cells lacking Mak16. These results demonstrate a strong genetic interaction between Mak16 and the CIA pathway, supporting the conclusion that Mak16 depends on an Fe/S cluster delivered via the CIA machinery. This synthetic lethality highlights the essential role of Fe/S cluster incorporation for Mak16 function and emphasizes the broader relevance of CIA-mediated Fe/S protein maturation in ribosome biogenesis.

### Destabilization of the Fe/S Cluster Coordination in Mak16 Disrupts Rpf1 Interaction.

The in vivo interaction between Mak16 and Rpf1 has been demonstrated through various methods, including high-throughput genetic studies (31), yeast two-hybrid assays (32), in vitro pull-down experiments (33) and cryo-EM structures (16–19, 34). Given the critical role of this interaction for ribosomal maturation, we investigated whether partial or complete abrogation of the Fe/S cluster coordination in Mak16 affects its association with Rpf1. Reciprocal immunoprecipitation in yeast cells depleted of chromosomally encoded Mak16 and Rpf1 (Gal-*MAK16*/Gal-*RPF1*) verified the interaction between plasmid-encoded HA-Mak16 and Myc-Rpf1 proteins (Fig. 5*B*). We then examined a set of Mak16 variants with differing degrees of Fe/S cluster disruption: a nonperturbing variant (C55A), a moderately affected variant (C38A), and two severely affected variants (C28A and C15A/C38A), based on our previous drop test and total RNA quantification data (Fig. 3 *D* and *E*). Both HA-Mak16 and Myc-Rpf1 protein levels were lower in extracts from cells expressing the more severely disrupted variants (C28A, C38A, and C15A/C38A). Notably, the degree of complex formation between Mak16 and Rpf1 correlated with the severity of growth defects (Fig. 3*B*) and decreased total RNA levels: the greater the disruption of Fe/S cluster coordination (i.e., 1 or 2 cysteine residues exchanged to alanine), the more severe the impairment of Mak16/Rpf1 association. These findings demonstrate that proper Fe/S cluster coordination is not only essential for Mak16 structural stability but also for its capacity to engage in functional interactions with Rpf1 during ribosome biogenesis.

### Destabilization of the Fe/S Cluster Coordination in Mak16 Increases Sensitivity to Redox Stressors.

Previous studies have shown that exposure of yeast cells to sublethal doses of reactive oxygen species (ROS) generating agents leads to cleavage of 25S rRNA, particularly in the c-loop of ES7 (35). In the experiments dithiothreitol (DTT) was also included, which, despite its common use as a stabilizing agent, by reaction with O_2_ increased the level of H_2_O_2_ in yeast (35). Interestingly, not ROS itself, but the presence of redox-active iron present in the ribosomes specifically contributed to destabilization of 25S rRNA through an unknown mechanism (36). The origin of free iron in the ribosomes was unclear, but was increased in cells lacking Grx5 (37). In this context, RNA cleavage was detected not only in 25S, but also observed in 18S, 5.8S, and 5S rRNA. Given that Mak16 harbors a redox-active [4Fe-4S] cluster and interacts extensively with ES7a, while being in proximity to ES7c, we tested the impact of destabilization of the Fe/S coordination in Mak16 on cells exposed to the redox stressors from the ES7 cleavage screen (H_2_O_2_, menadione, and DTT), supplemented by the superoxide generating compound paraquat. Since cells lacking Mak16 are nonviable, precluding the observation of any secondary phenotypes, we employ the sensitized Mak16 variant C38A, which already proved suitable in synthetic lethality assays with CIA depletion (Fig. 5*A*). Among the four agents tested, paraquat up to 20 mM had no discernible effect on growth of cells expressing wild type Mak16 or the C38A variant, with viability comparable to untreated controls (Fig. 5*C*). In contrast, DTT and hydrogen peroxide (H_2_O_2_) significantly decreased growth, indicating increased sensitivity to redox stress. Menadione elicited the strongest response causing a markedly greater effect than the other treatments. These results suggest that Mak16 and its C38A variant are vulnerable to ROS challenge, particularly quinone-induced stress. So we essentially reproduced a phenotype induced by the ROS agents previously described (35–37). In our cells, however, the observed growth effect was not related to an increased ferrous iron concentration in the cytoplasm but rather linked to the destabilization of the Fe/S cluster coordination in Mak16, potentially leading to release of free Fe^2+^ upon Fe/S cluster breakdown. The specific relationship between the observed cleavage sites (37) and iron released from the degradation of Fe/S on Mak16 remains, however, to be elucidated. A plausible explanation is that destabilization of the Fe/S cluster in Mak16 results in the release of free Fe^2+^, which may locally associate with rRNA. In an oxidative environment, this redox-active iron could facilitate site-specific RNA damage through Fenton-type reactions, particularly at exposed vulnerable structural regions such as ES7.

### Wild type Mak16 Is Sensitive to Oxidative Stress.

The growth phenotypes observed in sensitized cells (Fig. 5*C*) could, in principle, reflect effects on other essential Fe/S proteins or be specific to the C38A variant. To address this and capture the physiologically relevant situation in which all four cysteines coordinate the [4Fe–4S] cluster, we examined whether treatment with hydrogen peroxide, menadione, DTT, or paraquat affects the stability of wild type Mak16 and its interaction with Rpf1. DTT treatment did not alter Mak16 levels or Rpf1 binding (Fig. 5*D*), and paraquat likewise had no effect. In contrast, hydrogen peroxide and menadione caused a pronounced decrease in Mak16 abundance and in coprecipitated Rpf1, indicating destabilization of the Mak16–Rpf1 complex. The discrepancy between the mild growth phenotype and the lack of effect in pulldowns suggests that DTT may exert pleiotropic effects unrelated to Mak16. Although it is impossible to prove that H_2_O_2_ and menadione act solely via loss of Mak16 function to impair growth, the combined data (Fig. 5 *C* and *D*) strongly support the notion that Mak16 and the Fe/S protein Rli1 (38) are cytosolic Achilles’ heels of eukaryotes regarding oxidative stress.

To further investigate the intrinsic sensitivity of the Mak16 Fe/S cluster, we analyzed the purified ^57^Fe-enriched Mak16/Rpf1-Δ58 complex under defined stress conditions. Mössbauer spectroscopy revealed that DTT and menadione caused less than 5% loss of the [4Fe-4S]^2+^ cluster under anaerobic conditions, whereas hydrogen peroxide induced substantial cluster degradation, with formation of a [3Fe-4S]^1+^ species, confirmed by an EPR signal at *g* = 2.02 (Fig. 5 *E* and *F* and *SI Appendix,* Fig. S15). While menadione produces a strong phenotype in vivo, it had no direct effect on the Fe/S cluster in vitro, because in the absence of cellular redox systems, oxygen, and electron donors required for redox cycling, it cannot generate ROS and therefore cannot chemically react with the cluster. These findings indicate that menadione-induced damage in vivo arises entirely from intracellular ROS generation, rather than direct cluster destabilization. Taken together, these results establish that the wild-type Mak16 Fe/S cluster is intrinsically sensitive to oxidative stress. Hydrogen peroxide causes direct cluster breakdown, menadione highlights the contribution of intracellular ROS pathways, and the mild DTT effect observed in growth assays arises from secondary, indirect ROS formation. Paraquat has no measurable impact under either experimental context.

## Discussion

Our investigation into Mak16 was motivated by its consistent copurification with components of the CIA-targeting complex, which mediates the final delivery of Fe/S clusters to recipient proteins (20, 21). We were equally intrigued by the observation that a yeast allele leading to a change of a conserved cysteine residue (C38Y) of Mak16 exhibited defects in 60S ribosomal subunit maturation (39). Nevertheless, subsequent cryo-EM analyses of Mak16 in yeast preribosomal particles either lacked a metal cofactor (16, 18) or showed a Zn^2+^ ion (17, 19). More extensive electron density was reported only in human Mak16 at higher resolution, where it was provisionally modeled as an Fe/S cluster, though without validation (34). In this study, we present robust evidence that yeast Mak16 is a bone fide [4Fe-4S] protein. We further characterized the biophysical properties of both yeast and human Mak16, and define the in *vivo* significance of its Fe/S cluster in yeast Mak16 in the context of ribosome biogenesis.

To directly address the apparent discrepancies in metal cofactor assignment and to establish the relevance of an Fe/S cluster in Mak16, we employed the gold-standard ^55^Fe radiolabeling assay to assess the incorporation of ^55^Fe into affinity-tagged HA-Mak16 (Fig. 1*A*). Although ^55^Fe binding alone does not definitively establish the presence of a physiologically relevant Fe/S cluster, this observation, combined with the strict dependence on Nfs1, the essential mitochondrial cysteine desulfurase of the iron–sulfur cluster (ISC) assembly machinery, provides compelling evidence for an Fe/S cluster of Mak16 in vivo. Furthermore, efficient ^55^Fe incorporation into Mak16 required the CIA components Nar1 and Cia2, underscoring the importance of both the ISC and CIA pathways in its maturation. These results collectively demonstrate that Mak16 is a genuine Fe/S protein whose cluster biogenesis depends on the canonical cellular Fe/S machineries.

To further explore the phylogenetic conservation of Fe/S clusters in Mak16, we expanded our analysis to include two unicellular eukaryotic parasites with highly divergent cellular and metabolic adaptations: *T. brucei* and *E. cuniculi*. We demonstrated that Mak16 from both organisms, when expressed in yeast, also harbors an Fe/S cluster (Fig. 1*B*). In *T. brucei*, the causative agent of African trypanosomiasis (sleeping sickness), Mak16 thus appears to be an important client of both the ISC and CIA machineries, highlighting the essential role of Fe/S cluster biogenesis in this pathogen (40–42). Interestingly, tryptophan is the C-terminal amino acid residue of Mak16 proteins from *T. brucei*, *E. cuniculi*, and nearly all other trypanosomal and microsporidial species (*SI Appendix,* Fig. S16). Analysis of 1,900 eukaryotic Mak16 proteins reveals that 17% has tryptophan as C-terminus, a 14-fold enrichment when corrected for the abundance of tryptophan. The tryptophan is part of the consensus sequence [LIM]-[DES]-[WF], a motif known to recruit the CIA targeting complex in approximately 25% of all eukaryotic cytosolic and nuclear Fe/S proteins (43). In contrast, both yeast and human Mak16 lack this sequence, suggesting that in these organisms Mak16 may rely on an internal recognition sequence or employs a dedicated adaptor protein for recruiting the CIA targeting complex. Although the involvement of the CIA machinery in Mak16 maturation is established, several key questions remain, including whether Mak16 interacts with the CIA targeting complex only before and during cluster assembly, how its release is coordinated, and whether cluster insertion occurs prior to or following nuclear import.

We focused then on the biophysical properties of recombinant yeast and human Mak16 proteins (Fig. 2). Comprehensive characterization of human Mak16 and the yeast Mak16/Rpf1-Δ58 complex using EPR and Mössbauer spectroscopy revealed a [4Fe-4S]^2+/1+^ cluster in Mak16. The EPR and Mössbauer features of Mak16 closely resemble those observed for tetracysteinyl coordinated protein-bound [4Fe-4S]^2+/1+^ clusters (28, 44). EPR-monitored redox titrations in solution demonstrated that the [4Fe-4S]^2+/1+^ cluster exhibits a low midpoint potential of approximately –520 mV vs. NHE (Fig. 4 *A* and *B*). This potential is lower than that of [4Fe-4S] clusters of the bacterial ferredoxin type with typical midpoint potentials between –480 and –400 mV vs. NHE (45, 46). A key question was whether recombinant purified Mak16/Rpf1-Δ58 complex could bind its 25S rRNA interaction sequences, specifically the expansion segment 7 (ES7), a 210-nucleotide sequence that, according to high-resolution cryo-EM, adopts a characteristic L-shaped fold (16–19). This structure facilitates interaction with the Mak16 (or Nsa1) module, comprising Mak16, Rpf1, Nsa1, and Rrp1, clamping rRNA domains I and II during 60S ribosome assembly. EMSA analysis demonstrated stoichiometric binding between the Mak16/Rpf1-Δ58 complex and synthetic ES7 (Fig. 4*C*), establishing a critical prerequisite for subsequent evaluation of the redox properties of yeast Mak16 upon RNA binding. Under the conditions used for EMSA, the Mak16/Rpf1-Δ58/ES7 complex exhibited, if any, only a 20 mV upshift of the midpoint potential in solution as revealed by EPR spectroscopy (Fig. 4*D*). Such minimal shift cannot cause a relevant change of ES7 binding to the Mak16/Rpf1 complex at the prevalent glutathione and NADP(H) based potential of ~–300 mV in the eukaryotic cell (47). Therefore, Mak16 functions in ribosome biogenesis with its cluster in the [4Fe-4S]^2+^ state, which we consider to be its biologically active form. In contrast, studies on replicative polymerases and other DNA-interacting proteins have demonstrated that their binding to DNA induces a significant redox potential shift in their [4Fe-4S] clusters (48). Notably, these studies employed DNA-modified surfaces for cyclic voltammetry and are fundamentally different from electrochemical methods in bulk solution. Taken together, our results indicated that, while the interaction with ES7 is crucial for Mak16 function, the redox state of the Fe/S cluster does not modulate ES7 binding.

On other hand, our data demonstrated that the solvent-exposed [4Fe-4S] cluster of Mak16 is highly susceptible to breakdown under aerobic conditions or oxidative stress. Upon breakdown ferrous iron (Fe^2+^) is released, which via Fenton chemistry can generate hydroxyl radicals (^•^OH) capable of not only damaging DNA, but also RNA (49). Under redox stress, yeast expressing the C38A variant showed stronger growth defects than wild-type, indicating that 25S rRNA maturation requires an intact Fe/S cluster. Moreover, this finding mirrors the phenotype of the *mak16-1* allele described by Pellett and Tracy (39), in which substitution of Cys38 with tyrosine impaired 60S ribosome biogenesis. We therefore examined the functional consequences of cysteine variants of Mak16 in yeast cells. In the variants of the Fe/S cluster coordinating cysteine residues, levels of both Mak16 and its binding partner Rpf1 were decreased (Fig. 5*B*). Importantly, the cellular level of Mak16 correlated with the severity of observed phenotypes, including defects in cell viability, decreased rRNA synthesis, and disruption of the Mak16/Rpf1 complex. Phenotypic outcomes ranged from unaltered growth for the C15A variant, to moderate growth defects for C38A, and to complete growth arrest in C28A, C43A, and C15A/C38A variants (Fig. 3*B*). The total RNA yields, largely composed of rRNA, mirrored this gradient of severity (Fig. 3*D*). These results indicate the following scenario: disruption of Fe/S cluster coordination compromises Mak16 stability, impairs binding to Rpf1, leading to defective ribosome biogenesis, and ultimately loss of cell viability. Why is the Fe/S cluster of Mak16 so critical for 60S ribosomal subunit maturation? Cryo-EM structures have shown that the NTD of Mak16 containing the cluster binding cysteine residues is positioned at the core of a ribonucleoprotein pocket of the 25S rRNA hairpins 45 and 46, and the ribosomal proteins L4 and L32 (16, 18). The central domain of Mak16 is in close contact with ES7 and engages the Brix-domain-containing assembly factor Rpf1, which also contacts the basal part of ES7. Given that the Fe/S cluster is embedded within the NTD, its disruption likely induces severe conformational changes of Mak16 compromising the coordinated interactions with Rpf1 and rRNA. Consequently, destabilization or disruption of the Fe/S cluster impairs early 60S ribosomal subunit maturation.

To assess the consequences of impaired Fe/S cluster biogenesis of Mak16, we quantified total RNA levels in yeast strains depleted of Nfs1, Nar1, and Cia2, essential components of the ISC and CIA pathways. Depletion of these factors led to a significant decrease in total rRNA levels, with the 25S rRNA exhibiting the most pronounced reduction (Fig. 1 *C* and *D*). The consistent phenotypic severity of Mak16 cysteine variants (Fig. 3 *B* and *D*), which mimic the effects of impaired Fe/S cluster biogenesis, strongly indicate that the observed defects are specific to Mak16. This link was reinforced by synthetic lethality observed in strains expressing the C38A Mak16 variant in combination with depletion of the CIA components Cfd1, Nbp35, and Cia1 (Fig. 5*A*). Together, these findings underscore the essential role of the Fe/S cluster assembly machinery in maintaining Mak16 function, coordinating ribosome biogenesis, and ensuring cell viability. Extending beyond established roles in genome maintenance and translation (2), our findings now implicate Fe/S cluster biogenesis in one of the most fundamental steps in protein synthesis: the maturation of the large ribosomal subunit.

Building on the crucial role of Fe/S cluster biogenesis in ribosome maturation, it is important to consider the impact of redox stress on Mak16 function. Given its sensitivity (Figs. 2*E* and 5 *C*–*E*), it is plausible that Mak16 is an “Achilles’ heel” under oxidative stress conditions. This terminology has been applied to the Rli1 protein, which possesses two [4Fe-4S] clusters highly sensitive to oxidative stress (38, 50, 51). Current studies describe translation termination and ribosome recycling as primary functions of Rli1 (5). Rli1 (ABCE1 in humans) also functions in the “test driving” of the pre-40S or pre-60S particles in a cytoplasmic translation-like quality control step (52), but not directly in ribosomal maturation per se (34, 53). This mechanism echoes observations in mitochondrial translation, where the solvent-exposed [4Fe-4S] cluster of the mitoribosomal assembly factor METTL17 is destabilized by oxidative stress, leading to cluster loss and arrested mitoribosomal translation (9, 54). Not only the Fe/S cluster of METTL17, but also two of the three structural [2Fe-2S] clusters of the human mitochondrial ribosome (7, 8) are highly sensitive to H_2_O_2_ and their disruption impairs mitochondrial translation (54, 55). Mak16, Rli1, and the mitoribosomal Fe/S proteins underscore a conserved principle: even when Fe/S clusters do not mediate redox chemistry, they supply a redox sensing capability which allows to sense oxidative stress and attenuate translation. In conclusion, we identify Mak16 as a redox-sensitive Fe/S protein essential for 60S ribosomal subunit maturation. Our findings show that its [4Fe-4S] cluster, synthesized through the ISC and CIA pathways, plays a critical role in stabilizing Mak16 and facilitating its complex formation with the partner protein Rpf1. We propose that the redox sensitivity of the cluster enables Mak16 to function as a sensor for redox stress conditions, modulating ribosome biogenesis.

## Materials and Methods

Detailed descriptions of yeast and *E. coli* strains, growth conditions, complementation assays, cloning and mutagenesis procedures, western blotting, ^55^Fe incorporation assays, and protein–protein interaction studies in yeast cells are provided in the *SI Appendix*. Additional methods for overexpression and purification, as well as UV-Vis, EPR and Mössbauer spectroscopic characterization of yeast and human Mak16, are also described in the *SI Appendix*. RNA-related experiments, including RNA isolation, Northern blotting, synthesis of ES7 RNA, and electrophoretic mobility shift assays (EMSAs), are likewise detailed in the *SI Appendix*. Statistical analyses and reproducibility measures were performed with GraphPad Prism 9. Graphical data are presented as mean ± SEM. Statistical significance was assessed using multiple comparisons one-way ANOVA with Šidák correction. Mössbauer spectra were simulated with Lorentzian lineshapes in Microsoft Office Excel 2019, and Vinda for spectra recorded with applied fields (56, 57). EPR spectra were simulated with Gstrain5 (58). Structural alignment was performed with BIOVIA Discovery Studio 2024.

## Supplementary Material

Appendix 01 (PDF)

## Data Availability

All data have been deposited at Zenodo (59). All other data are included in the article and/or *SI Appendix*.
