# Supplementary material for: The function of Mak16 in ribosome biogenesis depends on its [4Fe-4S] cluster
Source: Proc Natl Acad Sci U S A. 2025 Nov 13;122(46):e2513844122. doi: 10.1073/pnas.2513844122 (PMC12646323; doi:10.1073/pnas.2513844122)
Supplement: Supplementary file 1 — Appendix 01 (PDF) [file pnas.2513844122.sapp.pdf]

Supporting Information for

The function of Mak16 in ribosome biogenesis depends on its [4Fe-4S] cluster

Nadine Duppe, Lukas Knauer, Marc Hagebölling, Lena Langner, Martin Stümpfig, Volker Schünemann, Antonio J. Pierik and Daili J. Netz

Antonio J. Pierik and Daili J. Netz
Emails: pierik@rptu.de and dnetz@rptu.de

This PDF file includes:

Supporting text
Figures S1 to S16
Tables S1 to S13
SI References

Supporting Information Text

Material and Methods

Yeast Strains. *Saccharomyces cerevisiae* W303-1A (MATa, *ura3-1*, *ade2-1*, *trp1-1*, *his3-11,15*, *leu2-3,112*) was used as wild-type strain. Exchange of the endogenous promoter of yeast genes by galactose regulatable promoters was achieved by homologous recombination. PCR-based DNA fragments designed according to (1) were amplified with the oligonucleotides listed in Table S5 and transformed using the lithium acetate method (2). In Gal-*MAK16* the promoter region of *MAK16* was replaced by the *natNT2* cassette from pYM-N27 (1). The same was done for the double mutant strains Gal-*MAK16*/Gal-*CFD1* and Gal-*MAK16*/Gal-*CIA1* which previously had their *CFD1* and *CIA1* promoter regions replaced by the *HisMX6* gene from pFA6a-HisMX6 (3). For the Gal-*MAK16*/Gal-*RPF1* strain the nucleotide-upstream promoter region of *RPF1* in Gal-*MAK16* was replaced by an adapted pYM-N27 template in which the *natNT2* gene was replaced by the *HisMX6* gene from pFA6a-HisMX6 (3). For the Gal-*MAK16*/Gal-*NBP35* strain the nucleotide-upstream promoter region of *NBP35* in Gal-*MAK16* was replaced by the *HisMX6* gene from pFA6a-HisMX6 (3). All constructs were verified by PCR amplification of chromosomal DNA, sequencing of the purified PCR fragments (see Table S6 for primers) and phenotypic analysis of yeast cells transformed with appropriate plasmids. The yeast strains used in this work are summarized in Table S7.

Cloning and Molecular Biology. For plasmids expressing yeast Mak16 or Rpf1 the coding sequence of *MAK16* and *RPF1* was amplified by PCR from genomic DNA of the W303-1A yeast strain. Templates for PCR amplification of *MAK16* from other organisms were a human *MAK16* cDNA clone (MGC:57551, Source Bioscience, UK) and genomic DNA from *E. cuniculi* and *T. brucei*, donated by Prof. J. Lukeš (University of South Bohemia, Czech Republic). The centromeric yeast vectors pRS414-*MET25* and pRS416-*MET25* (4) were used as "empty vectors" or recipient of DNA fragments, and named 414-*MET25* or 416-*MET25* for simplicity. In some yeast vectors the sequences coding for 3×HA (abbreviated HA) or 1×Myc (Myc) tags were introduced at the 5' end of *MAK16* and *RPF1*, respectively. The *E. coli* strains used are listed in Table S8. Primers used for plasmid construction are listed in Table S9. Cysteine to alanine variants were generated by amplification of plasmid templates with primer designed according to Zheng *et al*. (5) (Table S10), followed by *Dpn*I digestion to remove the parental template and transformation into *E. coli*. All yeast plasmids are listed in Table S11, and *E. coli* plasmids in Table S12. All constructs were verified by Sanger sequencing.

Growth Complementation Tests. For drop tests, exponentially growing cells were cultured in SC medium supplemented with 2 % (w/v) galactose or 2 % (w/v) glucose and diluted to an optical density (OD) at 600 nm of 0.1, followed by sequential 10-fold dilutions. Aliquots of 5 µL of undiluted and sequentially diluted cell suspensions were spotted onto solid SC agar media with the appropriate auxotrophy markers and sugar. Photographs were taken after 48 h of incubation at 30 °C. For the tests with redox stressors yeast cells were cultured for 16 hours in SC medium containing 2 % (w/v) glucose and auxotrophic markers, diluted to an OD_{600nm} of 0.2 and grown until the cells reached an $OD_{600\,nm}$ of 0.4, when they were treated with the redox stressing agents at the indicated concentration in the legend for 2 hours. The cells were then washed and spotted onto SC-glucose agar plates, as previously described or used for preparation of immunopulldowns at the indicated concentration of redox stressor.

^{55}Fe Incorporation. Yeast strains (see Table S7) were transformed with 416-*MET25* plasmids encoding HA-Mak16 from yeast or other organisms (see Table S10). To investigate the effect of depletion of key components of the ISC and CIA machineries, yeast cells transformed with 416-*MET25*-HA-Mak16 (from yeast) were grown in SC medium supplemented with 2 % (w/v) galactose or 2 % (w/v) glucose and auxotrophic markers for 24 h at 30 ˚C. Cells were then washed and incubated in iron poor SC medium supplemented with appropriate carbon source and auxotrophic markers for 16 h. Cells were again washed and resuspended in iron poor SC medium supplemented with appropriate sugar and auxotrophic markers, subsequently 10 µCi of $^{55}FeCl_3$ was added and cells were incubated for 2 h at 30 °C. To test whether Mak16 from other organisms is capable to bind ^{55}Fe expression was in W303-1A cells in 2 % (w/v) glucose supplemented SC medium. Protein extracts were prepared using normalized cell mass (0.5 g wet weight) in TNETG buffer (10 mM Tris/Cl pH 7.4, 2.5 mM EDTA, 150 mM NaCl, 10% (v/v) glycerol, 0.5% (v/v) Triton X-100) and 2 mM (end concentration) PMSF, using glass beads. An aliquot (25 µL) of the clarified supernatant was TCA-precipitated for further Western blot analysis (see below and Table S13 for antibodies). Mak16 was immunodetected using antibodies against the HA-tag (1:1000 diluted). Rabbit polyclonal antibodies were employed to document the depletion of ISC and CIA proteins. The remaining 200-250 µL were incubated with HA-agarose beads (HA-probe (F-7) AC sc-7392 AC, Santa Cruz Biotechnology) for 1 h at 4 ˚C. The immunoprecipitates were washed three times with TNETG buffer and resuspended in 50 µl of ddH_2O. After addition of 1 ml of scintillation

cocktail the ^{55}Fe radioactivity associated with the beads was measured by scintillation counting with settings appropriate for ^{3}H (6).

Western Blot Analysis. Samples for Western blot analysis were subjected to standard SDS-PAGE and subsequently transferred to nitrocellulose membranes (0.45 µm) using a Trans-Blot Turbo Transfer System® (Bio-Rad). The membrane was blocked with a BSA solution for monoclonal antibody detection and with skimmed milk for polyclonal antibody detection. Following the blocking step, the membrane was washed and then incubated with appropriate secondary antibody HRP conjugates, as detailed in Table S13. The membrane was then treated with enhanced chemiluminescence (ECL) reagent (BioRad), prepared by mixing the chemiluminescent substrate with 30% (w/v) hydrogen peroxide at a 1:1 ratio. Signal detection was performed using either the Fusion SL (Vilber Lourmat) or Chemostar (Intas) imaging system. In the Western blot images positive signals (luminescence) are displayed as dark bands on a white background from the signal inversion during image processing.

Protein-Protein Interaction: Co-immunoprecipitation was performed using wild-type or cysteine-to-alanine variants of yeast Mak16 and Rpf1, without any treatment or under conditions of redox stressors at specified concentrations. The experiments utilized affinity tags fused to Mak16 (N-terminal HA) and/or Rpf1 (N-terminal Myc). To avoid interference from endogenous, non-tagged copies, the Gal-Mak16/Gal-Rpf1 strain was employed. Following transformation with 416-*MET25*-HA-Mak16 (WT or variants) and 414-*MET25*-Myc-Rpf1 plasmids, yeast cells were cultured for 40 hours in SC medium supplemented with 2% (w/v) glucose and appropriate auxotrophic markers. The expression driven by the *MET25* promoter was reduced by adding 0.5 mM methionine (final concentration). Cell extracts were prepared from 0.5 g of yeast cells (wet weight) using glass beads in TNETG buffer, as previously described. An aliquot (50 µL) was precipitated with TCA for Western blot analysis, while 200–250 µL of the extract was incubated with either anti-HA-agarose beads (using either the HA-probe antibody (F-7, sc-7392, Santa Cruz Biotechnology, Fig. 5B) or magnetic anti-HA beads (Pierce #88830, Fig. 5D)) or Myc-agarose beads (using Myc antibody (9E10, sc-40, Santa Cruz Biotechnology, Fig. 5B) or magnetic anti-Myc beads (Pierce #88842, Fig. 5D)) for 1 hour at room temperature. The beads were then washed three times with TNETG buffer and resuspended in 50 µL Laemmli sample buffer (125 mM Tris-HCl, pH 6.8; 10% (v/v) glycerol; 2% (w/v) SDS; 0.1% (w/v) bromophenol blue; 100 mM DTT). Samples were subjected to SDS-PAGE and Western blotting. Anti-HA antibodies (diluted 1:2000 in Fig. 5B and 1:1000 in Fig. 5D) or anti-Myc antibodies (diluted 1:2000 in Fig. 5B and 1:1000 in Fig. 5D) were employed for detection. Chemiluminescence detection was performed as previously described.

Heterologous Protein Expression and Purification. Human and yeast Mak16 proteins were toxic when expressed in *E. coli* BL21 cells, a problem that was solved by the use of the more tightly controlled T7 Express LysY/Iq *E. coli* strain. Wild type and cysteine to alanine variants of yeast His_6-Mak16 were co-expressed with Rpf1 in pETDuet-1. After transformation three colonies were used for a 100 ml overnight preculture in LB medium plus ampicillin (0.1 µg/ml medium) at 30 °C. Of this preculture a 2 % inoculum was cultivated in the same medium until an $OD_{600\ nm}$ of 0.3 was reached. Then the temperature was decreased to 20 °C. Cells were induced with IPTG (0.5 mM, end concentration) at an $OD_{600\ nm}$ of 0.5 and further incubated for 16 h. Cells were harvested, transferred to a Coy anaerobic chamber and resuspended into lysis buffer (50 mM NaH_2PO_4, 300 mM NaCl, 10 mM imidazole, pH adjusted to 8.0 with NaOH). From this point onwards all steps were carried out anaerobically. Cell lysates obtained by sonication were centrifuged at 48,000 × g for 20 minutes. The resulting supernatant was applied to a 5 mL pre-packed Ni-NTA Agarose column (Cube Biotech), pre-equilibrated with lysis buffer, using an Äkta Start chromatography system (GE Healthcare) according to the manufacturer's instructions. The column was washed with lysis buffer containing 20 mM imidazole, and subsequently eluted with the same buffer containing 250 mM imidazole. When indicated, NaH_2PO_4 was replaced with 50 mM sodium pyrophosphate ($Na_4P_2O_7$). Immediately following elution, proteins were desalted using a PD-10 desalting column (GE Healthcare) pre-equilibrated with buffer containing 50 mM NaH_2PO_4 or $Na_4P_2O_7$ and 300 mM NaCl, adjusted to pH 9.0. Eluted proteins were either analyzed immediately or aliquoted, snap-frozen, and stored at –80 °C. If necessary, samples were concentrated using a 30-kDa cut-off centrifugal filter unit (Sartorius) prior to storage for later use. Western blotting followed by immunodetection with anti-His monoclonal antibodies was used to reveal which protein band on SDS-PAGE corresponded to the His-tagged Mak16 protein. Protein determination by the microbiuret/TCA method, and determination of non-heme iron and acid-labile sulfur were according to Pierik *et al.* (7). Extinction coefficients were calculated from the observed absorbance divided by the millimolar protein concentration, as determined by the microbiuret/TCA method.

EPR Analysis and EPR-mediated Redox Titrations. Purified Mak16, Mak16/Rpf1 or Mak16/Rpf1-Δ58 complex in desalting buffer (see previous section) were prepared for EPR analysis in the as isolated state, after oxidization with potassium ferricyanide (1 mM, final concentration) and after reduction with sodium dithionite (2 mM, final concentration) for 2-3 min in the Coy glove box. After transfer into EPR tubes and capping, the samples were subsequently frozen with liquid nitrogen outside the glove box. The determination of the redox potential of Mak16/Rpf1 in the presence or absence of ES7 was carried out in an anaerobic tent, using a mediator mixture containing 40 µM (final concentration) safranin O, neutral red, benzylviologen and methylviologen in 50 mM NaH_2PO_4 or $Na_4P_2O_7$ (as indicated in the legends) and 300 mM NaCl. The titration vessel was constantly stirred, during which a silver/silver chloride redox combination microelectrode (InLab™, Mettler Toledo, +207 mV vs. the hydrogen electrode) recorded the solution potential. After addition of small aliquots of 0.2-2 mM sodium dithionite in desalting buffer the potential was allowed to stabilize before samples were withdrawn for freezing and subsequent EPR analysis. EPR spectra were recorded with a Bruker Elexsys E580 X band spectrometer equipped with an Oxford Instruments ESR900 helium flow cryostat. A modulation amplitude of 1.5 mT and frequency of 100 kHz were used. Simulation of EPR spectra used the Gstrain5 program with LabVIEW as the graphical interface supplied by Prof. W.R. Hagen, TU Delft, the Netherlands (8). For spin integration 11.5 mM $CuSO_4$ in 10 mM HCl and 2 M $NaClO_4$ was used.

Mössbauer spectroscopy. ^{57}Fe metal powder was dissolved in 8 M HCl, followed by the addition of an equimolar amount of trisodium citrate dihydrate. The resulting solution was slowly neutralized to approximately pH 5 by addition of 12.5 % (w/v) aqueous ammonia. For ^{57}Fe incorporation, *E. coli* cells harbouring the appropriate plasmid were grown in LB medium supplemented with 50 µM ^{57}Fe-ammonium citrate (final concentration), inoculated with 1% (v/v) of an overnight preculture. Protein overexpression was performed as described in the section *Heterologous Protein Expression and Purification*. Eluted protein fractions in desalting buffer were concentrated to a final volume of ~400 µL using centrifugal spin concentrators. Samples were transferred to Mössbauer sample vials within an anaerobic chamber, if applicable reduced by addition of buffered sodium dithionite, rapidly frozen, and stored in liquid nitrogen. Mössbauer spectra were recorded in the constant acceleration mode with a conventional spectrometer from Wissel GmbH with a bath cryostat (Oxford Instruments, Abingdon, UK). Isomer shifts are given relative to α-Fe at 25 °C. Magnetically split spectra were simulated with the spin Hamiltonian formalism (9) with the program Vinda (10). Otherwise, spectra were analyzed by least squared fits using Lorentzian line shapes. For fitting of experimental Mössbauer spectra with more than two components the parameters for the species observed in isolation (oxidized human Mak16 for the 25 % component in dithionite reduced human Mak16 in the lower trace in Fig. 2B, and $[3Fe\text{-}4S]^{1+}$ from a difference spectrum at the bottom right for the other spectra in Fig. S15) were fixed.

RNA Isolation and Gel Electrophoresis. Total RNA was isolated from *Saccharomyces cerevisiae* strains W303-1A, Gal-*NFS1*, Gal-*NAR1*, and Gal-*CIA2* cultivated in synthetic complete (SC) medium supplemented with 2% (w/v) glucose or galactose, as indicated, and appropriate auxotrophic markers. RNA was also extracted from Gal-*MAK16* cells previously transformed with the empty vector 416-*MET25* or with 416-NP-Mak16 constructs encoding either wild-type or cysteine to alanine variants of Mak16. Single colonies were cultured for 24 h at 30 °C in the indicated carbon source, diluted into fresh medium to an initial $OD_{600\,nm}$ of 0.1 and then grown for approximately two doubling times. Cells were rapidly cooled on ice, washed with ice-cold RNase-free water, and harvested by centrifugation at 4 °C (corresponding to 4 $OD_{600\,nm}$ units, ~6.8×10^7 cells). Pellets were stored at −80 °C until RNA extraction. Total RNA was purified using the Monarch Total RNA Miniprep Kit (New England Biolabs) according to the manufacturer's instructions. RNA concentration and purity were determined using a NanoDrop spectrophotometer (Thermo Fisher Scientific). When indicated, RNA integrity and size distribution were analyzed by 1 % (w/v) agarose, 2.2 M formaldehyde gel electrophoresis using 30 mM tricine/30 mM triethanolamine buffer as running buffer. Samples were mixed with RNA loading buffer containing 0.025% (w/v) ethidium bromide. RiboRuler High Range RNA ladder (Thermo Fisher Scientific) was used as a molecular weight marker.

Northern Blot Analysis. Total RNA was extracted from *Saccharomyces cerevisiae* cells using the E.Z.N.A.® Yeast RNA Kit (Omega BioTek) following the manufacturer's instructions. RNA samples were resolved by electrophoresis on a 1 % (w/v) agarose gel containing 10 mM sodium acetate, 1 mM EDTA, 40 mM MOPS, pH 7.0 and 0.4 M formaldehyde. Following separation, RNA was transferred onto Hybond-N+ membranes (Amersham, GE Healthcare) using a TurboBlotter Downward Transfer System (Cytiva) with 10 × SSC (1.5 M NaCl and 0.15 M sodium citrate, pH 7.0) as the transfer buffer. RNA was crosslinked to the membrane using a BLX-254 UV crosslinker (Vilber Lourmat) at 245 nm for 1 min. Transfer efficiency was confirmed by ethidium bromide fluorescence present in the RNA loading buffer, visualized during the crosslinking step. Hybridization was performed with DIG-ddUTP-labeled probes specific to 18S (5′-CATGGCTTAATCTTTGAGACC-3′) and 25S (5′-CTCCGCTTATTGATATGC-3′)

rRNA. Probes were hybridized for 1 h at 37 °C (18S) and 45 °C (25S) in hybridization buffer containing 0.5 M sodium phosphate (pH 7.0), 7 % (w/v) SDS, and 1 mM EDTA. Membranes were washed sequentially in 2 × SSC with 0.1 % SDS for 5 min at room temperature and then for 5 min at 68 °C. For immunodetection, membranes were equilibrated in 0.15 M NaCl, 0.1 M maleic acid buffer (pH 7.5), then blocked for 30 min in the same buffer with 10 % (w/v) blocking reagent (Roche). Membranes were incubated with anti-digoxigenin-peroxidase (anti-DIG-POD) antibody (1:1000 dilution) in blocking solution for 1 h. After washing, chemiluminescent signals were developed using Clarity ECL substrate (Bio-Rad) and imaged with a Fusion SL documentation system (Vilber Lourmat). In the Northern blot images positive signals (luminescence) are displayed as dark bands on a white background from the signal inversion during image processing.

Synthesis of ES7-RNA. For the synthesis of ES7 RNA a DNA sequence was PCR amplified from yeast genomic DNA with a forward primer containing the minimal T7 promotor sequence (TAATACGACTCACTATAGGG) fused to the 5'-ES7 encoding DNA sequence (CATTTGATCAGACATG), and a reverse primer corresponding to the 3' end of ES7 (GGCATATAACCATTATGCCAGCATC). The amplified PCR product was used as template for the synthesis of ES7 using the HiScribe T7 Quick High Yield RNA synthesis kit (NEB). The run-off transcript started with the previously underlined GGG sequence. After digestion of the DNA template with DNase I, the synthesized ES7 RNA was isolated using the Monarch clean up kit with high capacity columns (NEB). ES7 RNA concentrations were quantified with a Nanodrop spectrophotometer (Fisher Scientific).

EMSA. To assess whether the synthesized ES7 could bind to the purified His_6Mak16/Rpf1-Δ58 complex, an electrophoretic mobility shift assay (EMSA) was conducted. A folding procedure was tested for the isolated ES7 RNA (see previous section) by incubation at 85°C for 30 seconds, followed by a cooling phase from 85 to 30 °C at a ramp rate of 1.5 °C/min in a buffer containing 50 mM sodium pyrophosphate, 300 mM NaCl, 1 mM DTT, 1 mM $MgCl_2$, and 10 % (v/v) glycerol (binding buffer). As the mobility of the RNA on non-denaturing gels did not change, this procedure was omitted in later experiments. Mixtures of purified His_6Mak16/Rpf1-Δ58 complex and ES7 RNA were loaded onto a 5 % (w/v) native acrylamide gel and electrophoresed in 45 mM Tris, 45 mM boric acid, 1 mM EDTA buffer. RNA was visualized after a 30-minute incubation with GelRed (1:10000 dilution), followed by washing and detection using an imaging system (Chemostar, Intas or Fusion SL, Vilber Lourmat). The gel was subsequently stained with Coomassie Brilliant Blue to visualize proteins.

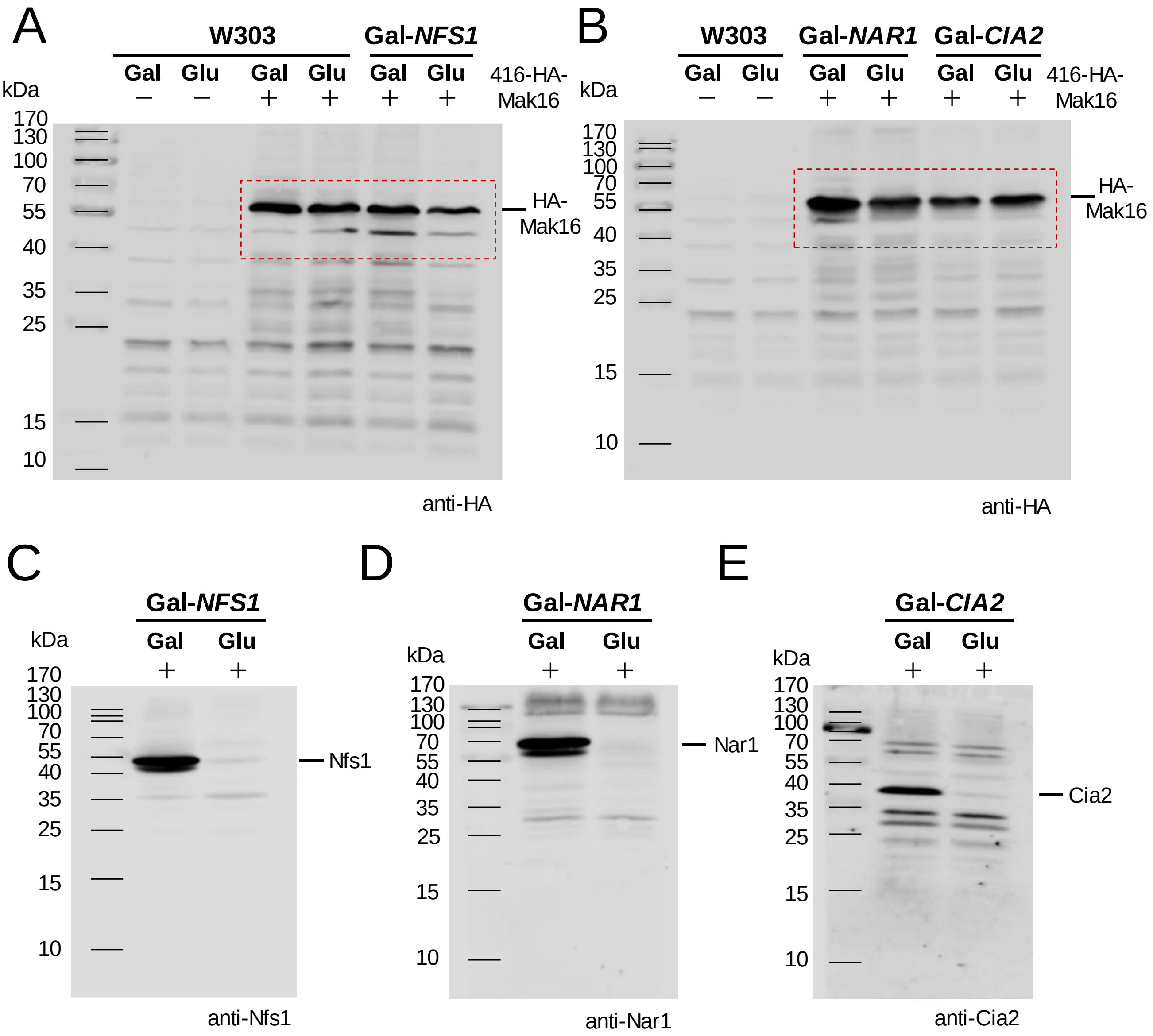

Fig. S1. Non-cropped images of Western blots shown in **Fig. 1A-B**. Red boxes depict the area of the images in the main text. Yeast wild type (W303) and the Gal-*NFS1* (**A**), Gal-*NAR1* and Gal-*CIA2* (**B**) strains transformed with 416-*MET25*-HA-Mak16 were grown under permissive (galactose, Gal) and non-permissive (glucose, Glu) conditions for 40 h in SC medium supplemented with the appropriate sugar. Cell extracts were prepared, subjected to SDS-PAGE and Western blotting. Commercial murine monoclonal antibodies against HA were used at a dilution of 1:1000, anti-mouse secondary antibodies at dilution of 1:10000. The efficiency of depletion of Nfs1 (**C**), Nar1 (**D**) and Cia2 (**E**) was assessed by Western blot analysis of the cell extracts as in (**A**) and (**B**) employing polyclonal antibodies raised against each protein in rabbits at a dilution of 1:1000 and anti-rabbit secondary antibodies at dilution of 1:10000.

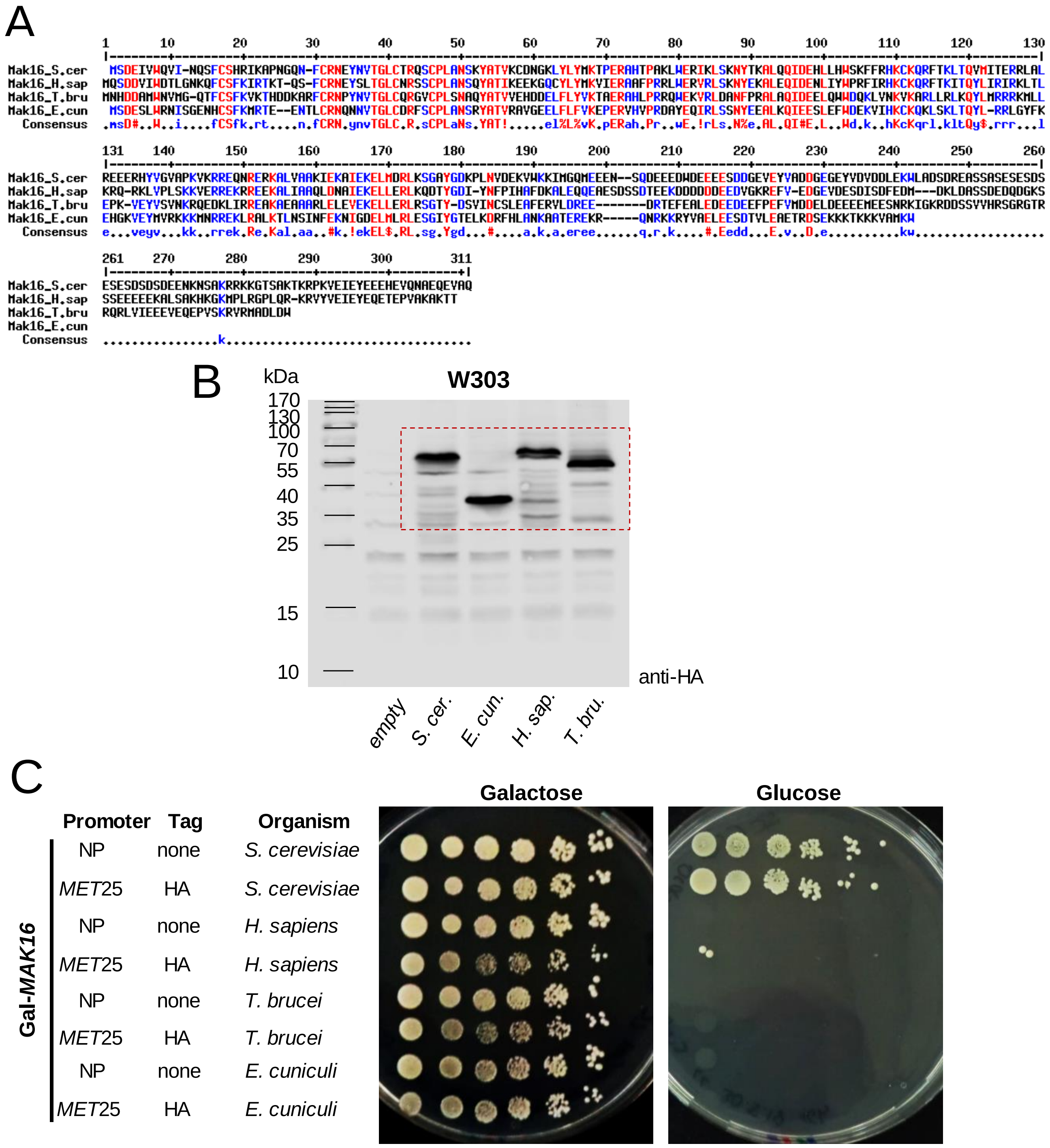

Fig. S2. (**A**) Amino acid sequence alignment (MultAlin (11)) between the sequences of Mak16 from *Saccharomyces cerevisiae*, *Homo sapiens*, *Trypanosoma brucei* and *Encephalitozoon cuniculi*, from top to bottom. (**B**) W303 yeast cells transformed with 416-*MET*25-HA-Mak16 from indicated eukaryotes were grown in glucose containing SC medium for 40 h, cell extracts were subjected to SDS-PAGE and Western blotting. Murine monoclonal antibodies against HA were used at a dilution of 1:1000, anti-mouse secondary antibodies at dilution of 1:10000. (**C**) Mak16 function in yeast cannot be substituted by tested homologues. The Gal-*MAK16* strain was transformed with 416 plasmids, containing yeast Mak16 or its homologues under the control of either the yeast *MAK16* promoter (NP) or the *MET25* promoter, as indicated. Cells were cultured in SC media containing galactose or glucose for 40 h, serially diluted 10-fold starting from an $OD_{600\ nm}$ of 0.5, and spotted onto plates. Growth was monitored for 2–3 days, with photographs taken at the end of the incubation period.

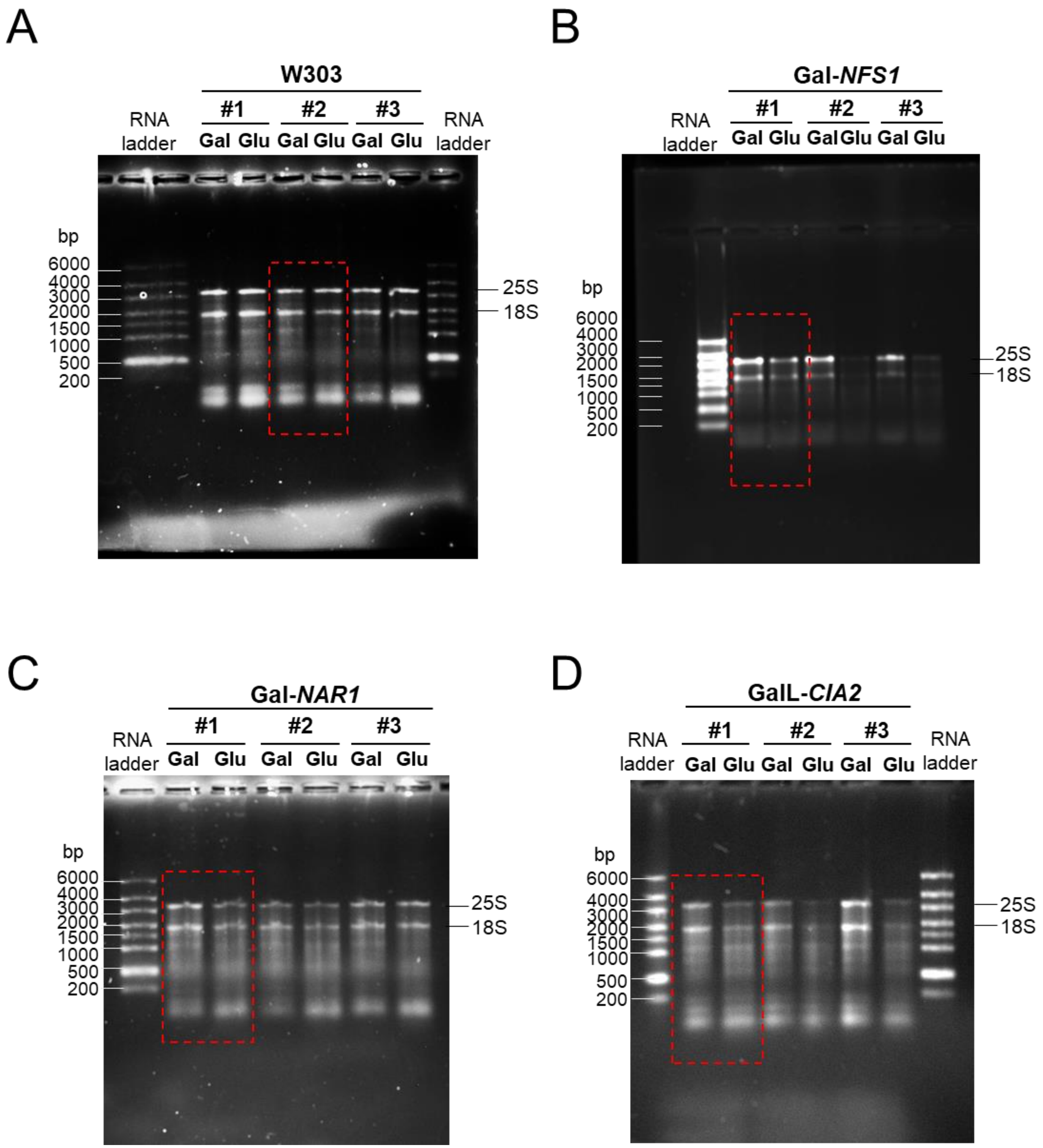

Fig. S3. (A-D) Non-cropped agarose gel electrophoresis showing total RNA extracted from the indicated yeast strains grown on galactose (Gal) or glucose (Glu), corresponding to the data in **Fig. 1D.** Data for three experiments (#1, #2 and #3) are shown. Gels were post-stained with GelRed and documented using ChemoStar Touch (Intas).

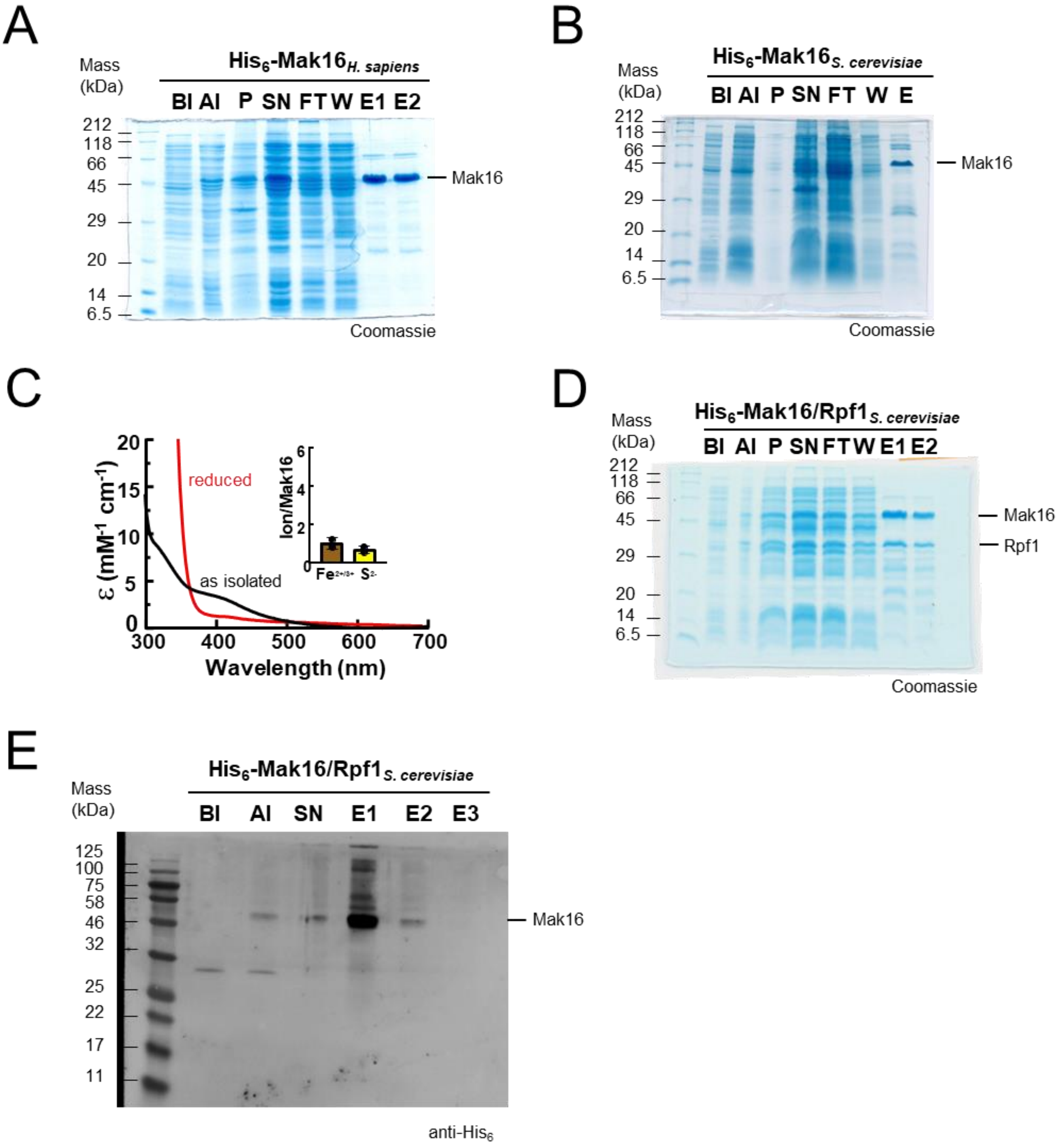

Fig. S4. (**A**) SDS-PAGE demonstrating the production and isolation of human His_6-Mak16 (data corresponding to **Fig. 2A**). (**B**) SDS-PAGE illustrating the expression and purification of yeast His_6-Mak16. (**C**) UV-Vis spectra of yeast His_6-Mak16 in its as isolated form (black line) and dithionite reduced form (red line), along with quantification of non-heme iron and acid-labile sulfide (inset). (**D**) SDS-PAGE for co-expression of yeast His_6-Mak16 with yeast Rpf1. (**E**) Western blot of co-expressed yeast His_6-Mak16/Rpf1, probed with a rabbit anti-His polyclonal antibody (1:1000 dilution) and anti-rabbit secondary antibodies at dilution of 1:10,000.

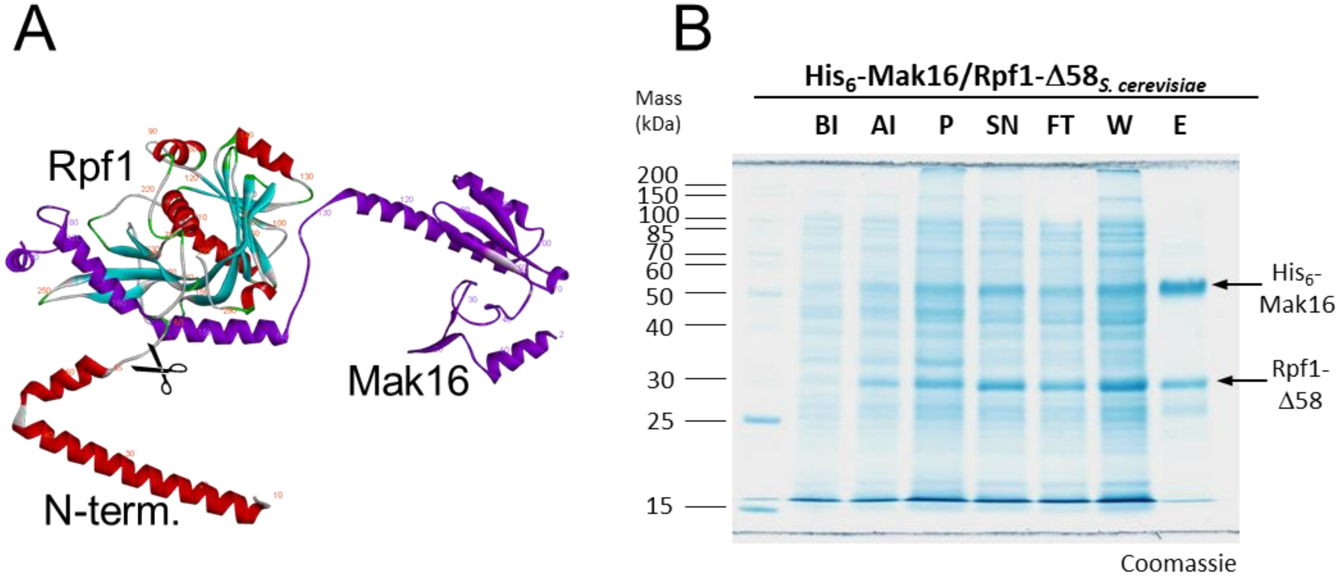

Fig. S5. (**A**) Visualization of the yeast Mak16-Rpf1 module (PDB ID: 6C0F). The truncation site, for removal of the N-terminal 58 amino acids of Rpf1 is marked with a scissors icon. The visualization, including the depiction of structural components, was created using BIOVIA Discovery Studio 2024. (**B**) SDS-PAGE analysis showing the overexpression and purification of yeast His_6-Mak16 co-expressed with Rpf1 truncated at its N-terminus to position 58.

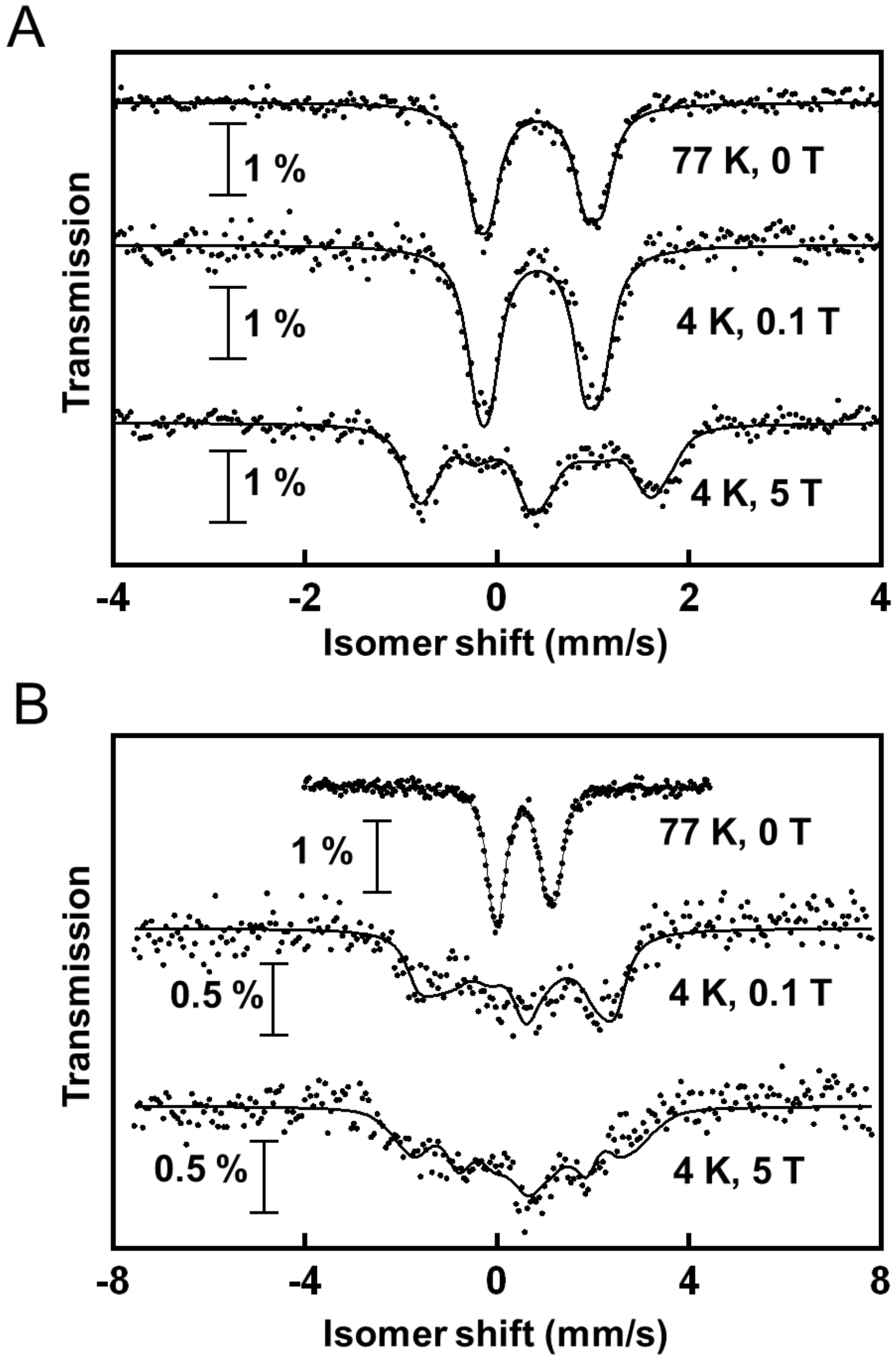

Fig. S6. Mössbauer spectra of the anaerobically purified yeast His-Mak16/Rpf1-Δ58 complex in 50 mM sodium phosphate buffer, 300 mM NaCl, pH 8.0 (**A**) and after incubation with sodium dithionite (4 mM, final concentration) for 2 min. (**B**). For Mössbauer parameters see Table S1. The top traces in both panels are identical to the experimental spectrum with the fit to the experimental data (solid trace) at 77 K as shown in the main text, **Fig. 2 F**, as the top and bottom trace, respectively. The middle and lower trace is for the same sample recorded at 4 K at an applied magnetic field parallel to the γ rays of 0.1 T and 5 T, respectively. In (**B**) spectra were recorded at a broader range of velocities to reveal splitting due to the paramagnetism of the *S*=1/2 cluster. The solid traces are fits based on Mössbauer parameters for the $[4Fe\text{-}4S]^{1+}$ cluster of *Bacillus stearothermophilus* ferredoxin (Table 2 in (12)).

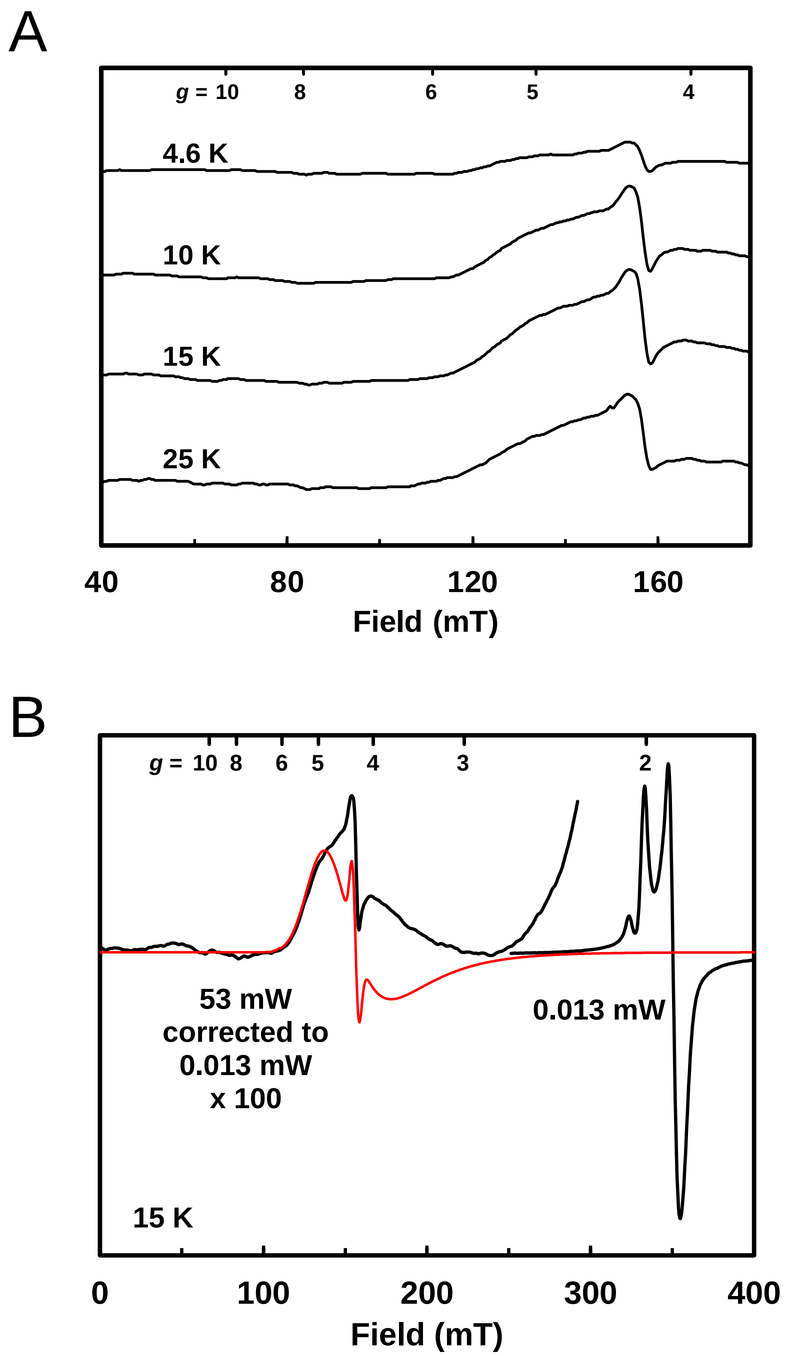

Fig. S7. EPR spectra of purified yeast His-Mak16/Rpf1-Δ58 complex in 50 mM sodium phosphate buffer, 300 mM NaCl, pH 9.0 after incubation with sodium dithionite (4 mM, final concentration) for 2 min. (**A**) Temperature dependence of the high spin spectral features. The intensities were corrected for a Curie law dependence, i.e. the *g*=4.4 signal is from an excited state. EPR conditions: microwave power, 50 mW, modulation amplitude, 1.5 mT, microwave frequency, 9.352 GHz. (**B**) Simulation (red) assuming that signal derives from the |±½⟩ doublet as excited state of a *S*=3/2 system with parameters as in Table S3. The low field region was recorded under conditions as in (**A**), but the intensity was divided by 64 (to correct the amplitude to a microwave power of 0.013 mW) and amplified 100-fold. The *g*=2 region was recorded at a microwave power of 0.013 mW.

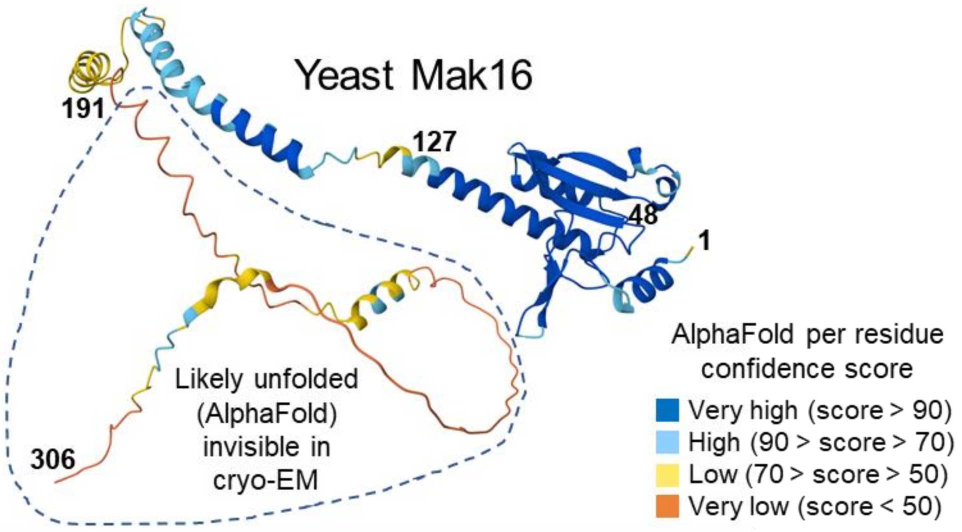

Fig. S8. AlphaFold model of yeast Mak16 with labelled positions of domains. Position 1-191 has defined electron density in the cryo-EM structure, position 192-306 is not visible.

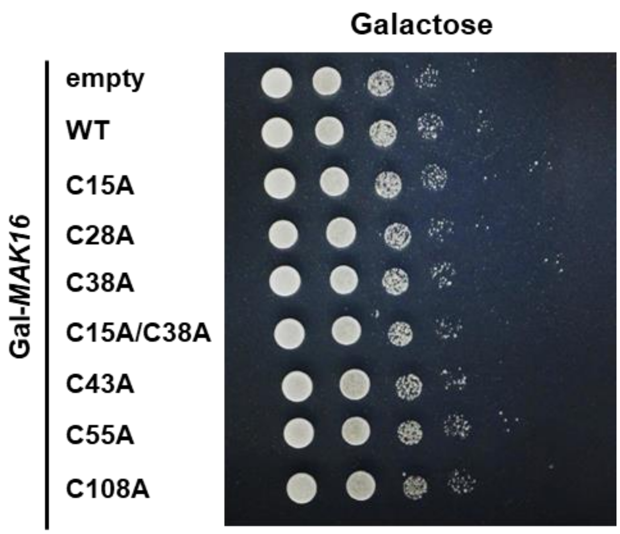

Fig. S9. Drop tests for Gal-*MAK16* yeast cells, transformed with 416 plasmids under control of the *MAK16* promoter under permissive conditions. Cells transformed with a plasmid lacking an insert (empty), or encoding wild type (WT) or indicated cysteine variants of Mak16, were cultured for 16 h in galactose containing SC medium. OD-normalized 10-fold serial dilutions were spotted on agar plates of the same medium and photographed after 2 days at 30 °C.

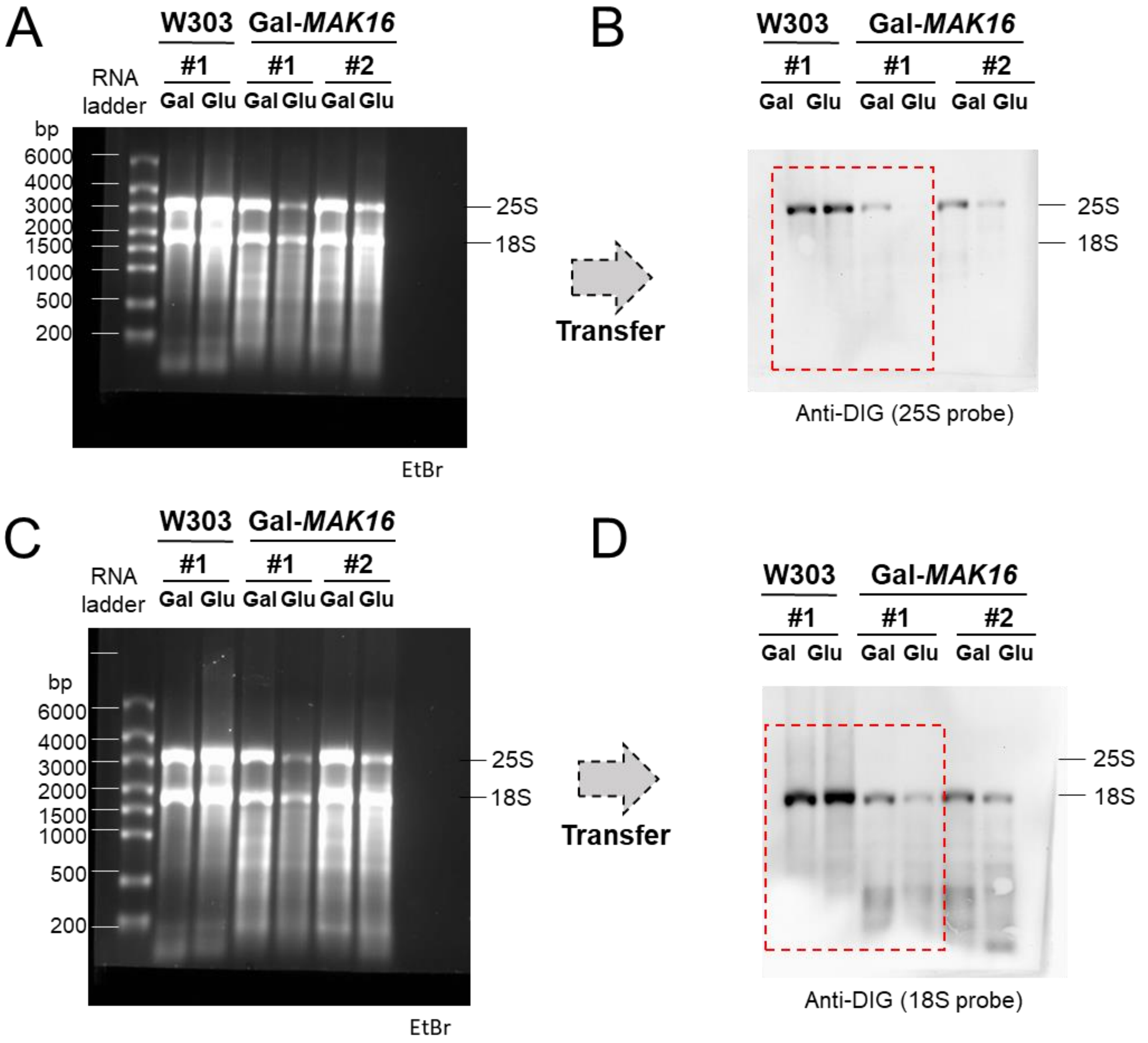

Fig. S10. Ethidium bromide visualization of total RNA isolated from wild type (W303) and Gal-*MAK16* yeast cells cultivated for 40 h in SC medium containing galactose (Gal) or glucose (Glu) subjected to agarose electrophoresis. After documentation by transillumination ((**A**) and (**C**)), RNA was transferred to a nylon membrane. In these Northern blots 25S or 18S rRNA was visualized with digoxigenin-ddUTP labelled probes and anti-digoxigenin-peroxidase conjugate ((**B**) and (**D**)). Marked areas correspond to the data shown in **Fig. 3C**.

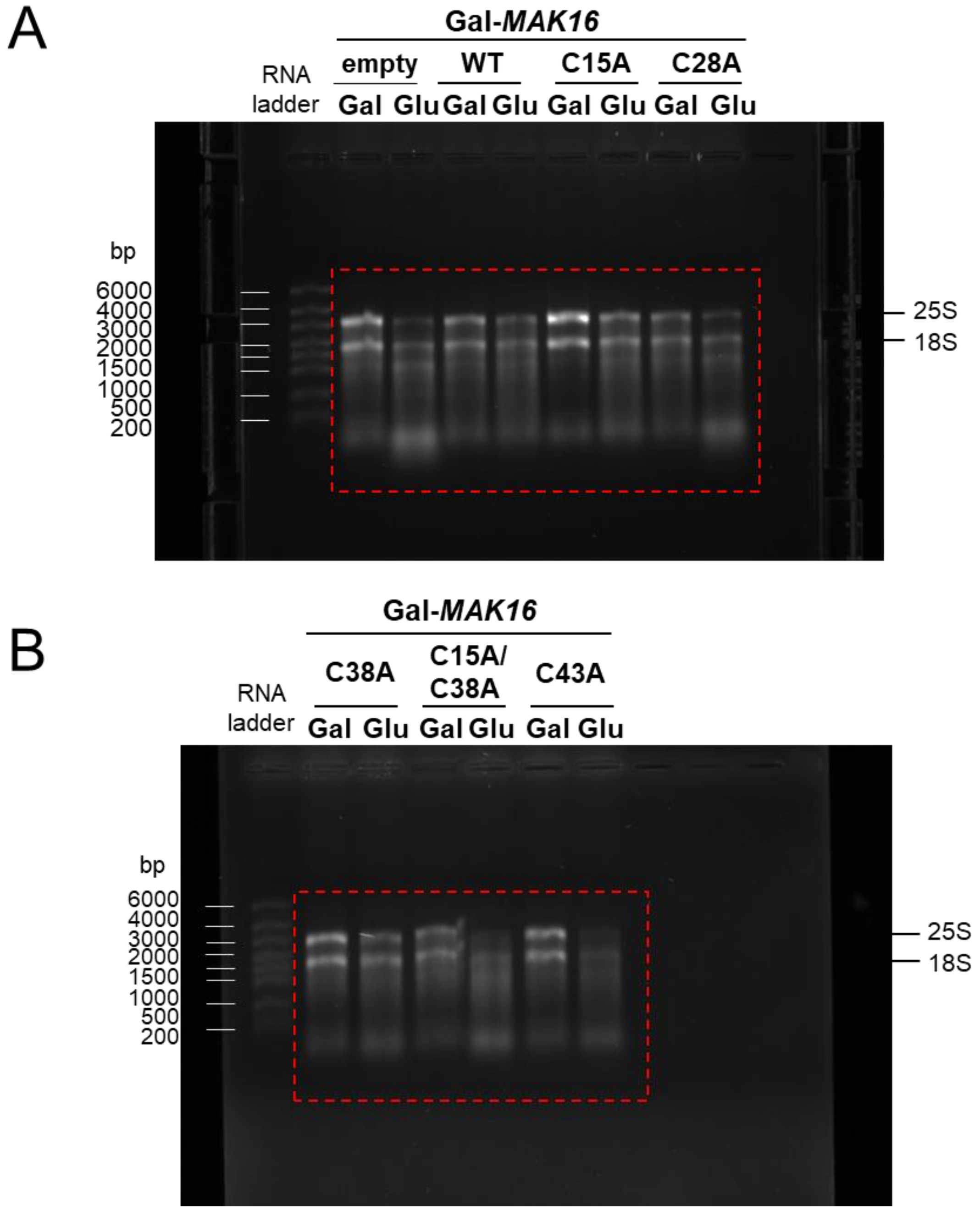

Fig. S11. (A-B) Uncropped agarose electrophoresis gels showing total RNA extracted from Gal-*MAK16* yeast cells transformed with empty vector, or 416NP vectors encoding wild-type and cysteine variants of Mak16. Growth was in galactose (Gal) or glucose (Glu) containing SC media. Gels were post-stained with ethidium bromide and documented using ChemoStar Touch (Intas). Marked areas correspond to the cropped data in **Fig. 3E**.

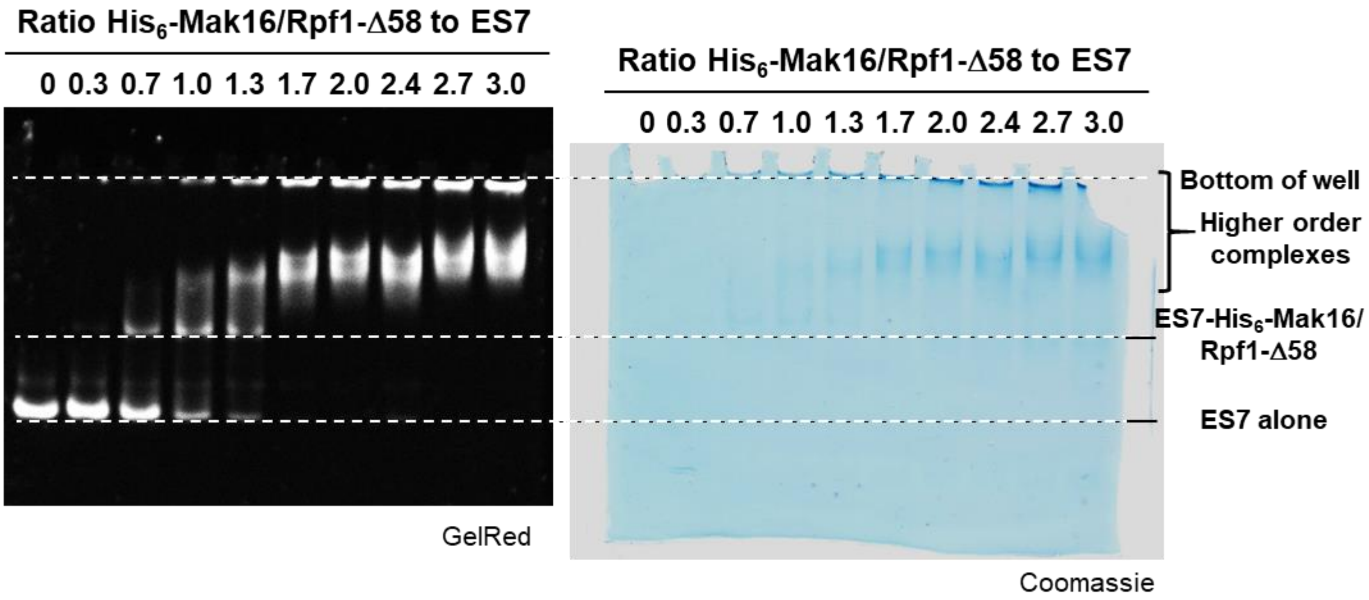

Fig. S12. Coomassie stained gel (right) after 5 % non-denaturing acrylamide gel electrophoresis and ChemoStar Touch (Intas) documentation of RNA with GelRed (left, **Fig. 4C).** The photograph and gel documentation are aligned to show which RNA bands correspond to RNA-Mak16//Rpf1-Δ58 complexes at different His_6Mak16/Rpf1-Δ58 to rRNA (ES7) ratios.

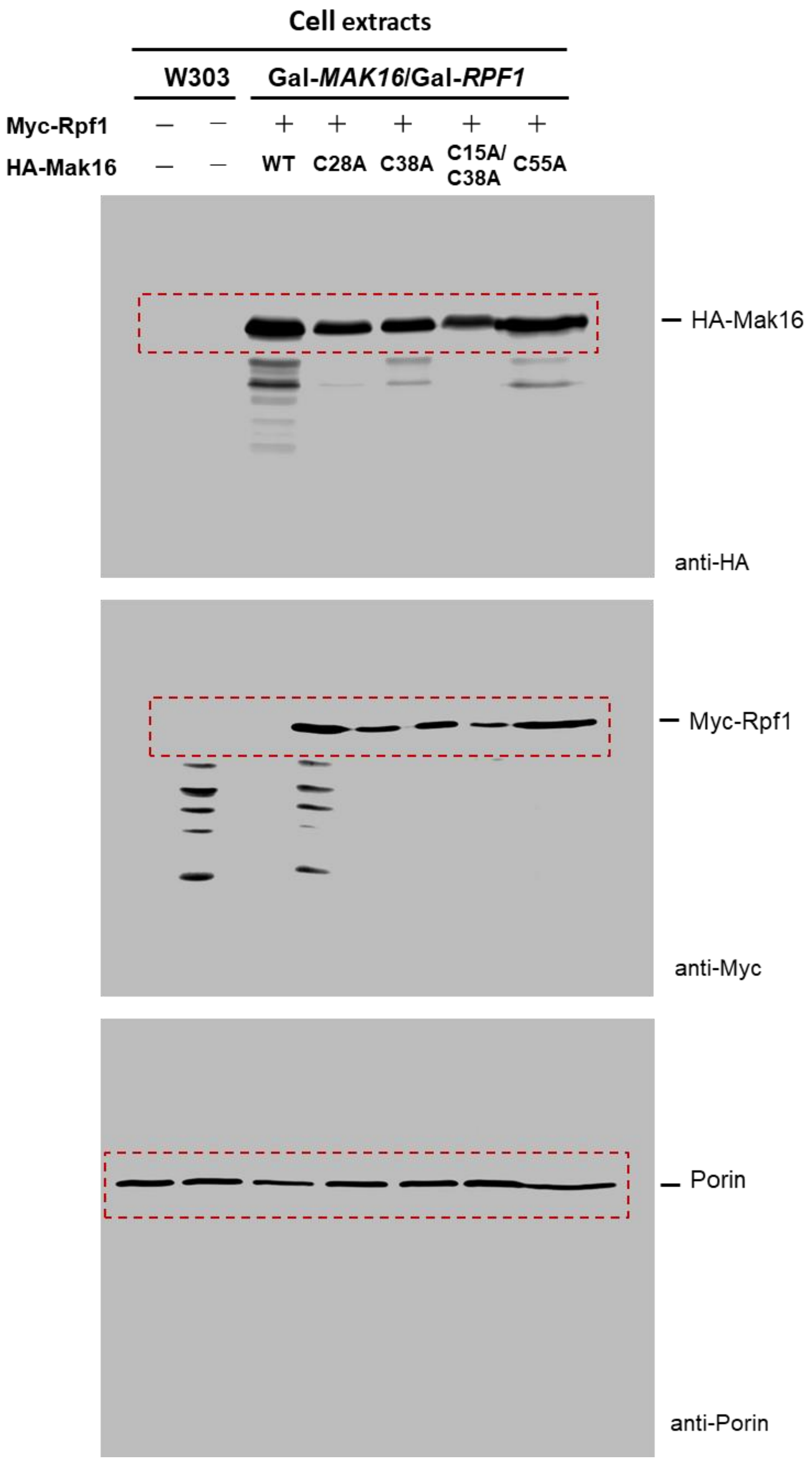
Cell extracts
W303
Gal-MAK16/Gal-RPF1
Myc-Rpf1 – – + + + + +
HA-Mak16 – – WT C28A C38A C15A/ C38A C55A
HA-Mak16
anti-HA
Myc-Rpf1
anti-Myc
Porin
anti-Porin

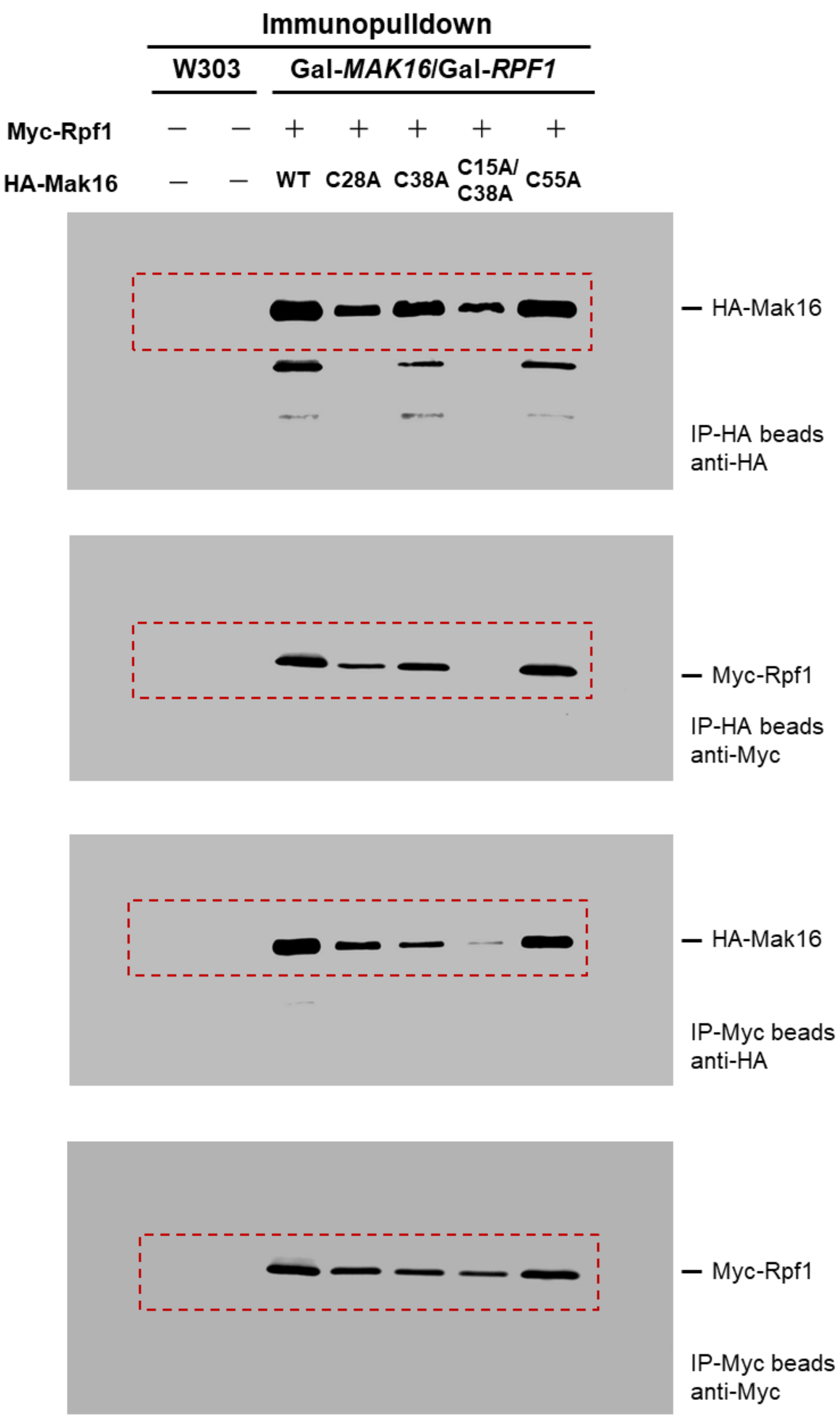

Fig. S13. Non-cropped images of Western blots shown in **Fig. 5B**. Red boxes mark the areas of the images in the main data. Polyclonal rabbit antibodies raised against porin (dilution 1:1000), primary monoclonal antibodies against HA and Myc tags were used at a dilution of 1:2000 and secondary anti-rabbit IgG antibodies at dilution 1:10,000.

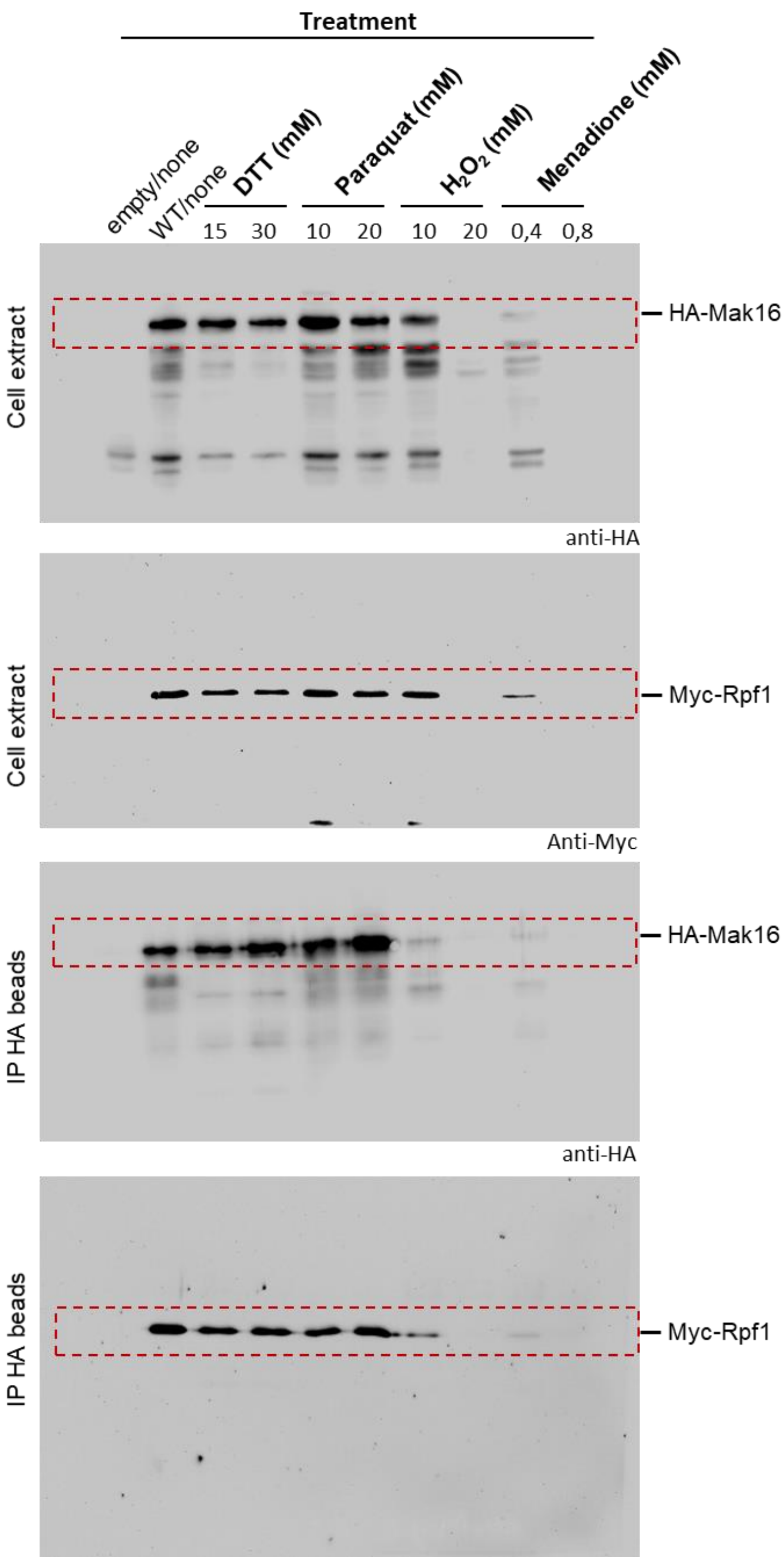

Fig. S14. Non-cropped images of Western blots shown in **Fig. 5D**. Red boxes mark the areas of the images in the main data. Monoclonal antibodies against HA and Myc tags were used at a dilution of 1:1000; anti-rabbit secondary antibodies were used at 1:10,000 dilution.

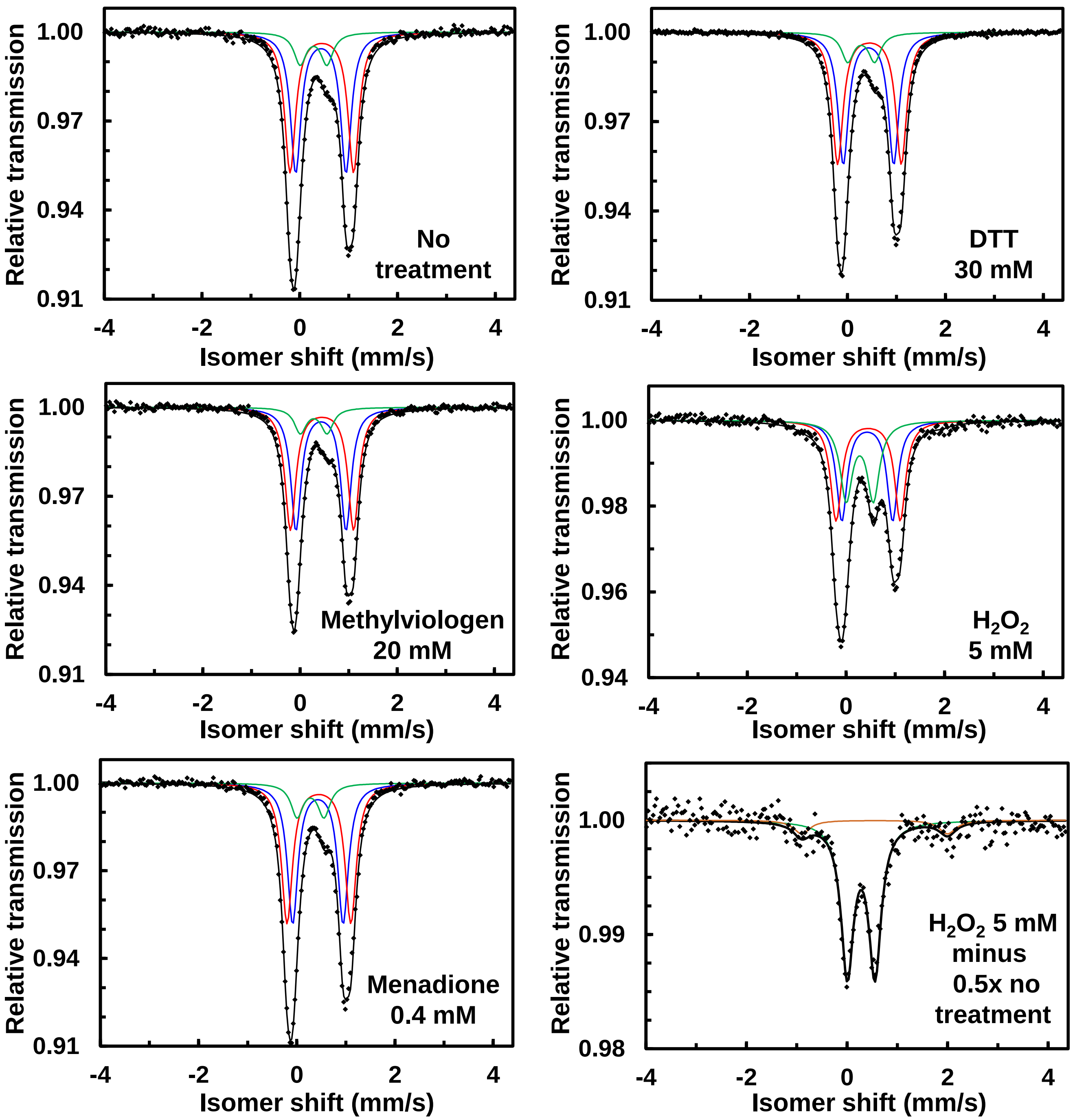

Fig. S15. Mössbauer spectra recorded at 77 K of ^{57}Fe enriched purified yeast His-Mak16/Rpf1-Δ58 complex in 50 mM sodium phosphate buffer, 300 mM NaCl, pH 9.0 after treatment with indicated components for 30 min. at 23 °C. Simulations are for the $[4Fe\text{-}4S]^{2+}$ quadrupole doublets (red and blue) and a quadrupole doublet for $[3Fe\text{-}4S]^{1+}$ (green). Mössbauer parameters in Table S1. The lower intensity of the sample treated with 5 mM H_2O_2 was due to non-optimal positioning of the sample in the cryostat. At the bottom right the difference spectrum for the determination of the Mössbauer parameters for the $[3Fe\text{-}4S]^{1+}$ and a minor ferrous component (brown) is shown.

A

**C-terminus of Mak16**	**Organism**
EEEVEQEPVSKRVRTADLDW	*Trypanosoma brucei brucei* TREU927
EEEVEHEHAPKRMRTADLDW	*Trypanosoma congolense* IL3000
IEEEVEHEQQKRSRLDDLDW	*Trypanosoma cruzi strain* CL Brener
EEEVEREQGTKRARLDDMEW	*Trypanosoma vivax* Y486
EEVERELPPRQRSRLEDLEW	*Leishmania braziliensis* MHOM/BR/75/M2904
EEVEQELAPRQRARLEDLEW	*Leishmania tarentolae*
EEVERELAPRQRARLEDLEW	*Leishmania major strain* Friedlin
DTTKSRKKSKSKKKSKQLEW	*Enterocytozoon bieneusi* H348
EAETRDSEKKKTKKKVAMKW	*Encephalitozoon cuniculi* GB-M1
ESCGVEKKADKKTKKTAMKW	*Encephalitozoon intestinalis* ATCC 50506
YEKEEEFYPLDRTHKISWDW	*Theileria annulata*
IEEEIENEPLKRVRQEDLDW	*Angomonas deanei*
LEEEVEREPRQRLRPEDIEW	*Leptomonas pyrrhocoris*
IEEEVEHEAPKRARREELDW	*Strigomonas culicis*
LEEEVEREPLQRARPEDLDW	*Crithidia fasciculata*
EREVEYDSKAKKTQRDELDW	*Bodo saltans*
PYVEIEYEQEQETANGDLNW	*Phytophthora infestans* T30-4
HIEYEQERENEKIPAVQHSW	*Schizosaccharomyces pombe*
SVEVEYEEDNGEKEKQMVDW	*Yarrowia lipolytica* CLIB122

B

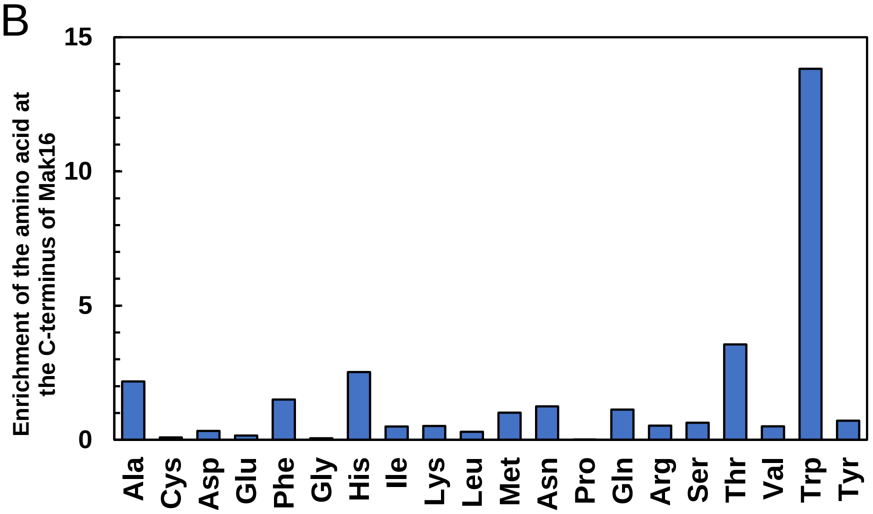

Fig. S16. (A) Tryptophan occurs at the C-terminus of Mak16 in eukaryotes, especially in many trypanosomal and microsporidial species. **(B)** Enrichment of tryptophan at the C-terminus in a collection of 1900 eukaryotic full-length Mak16 proteins. The percentage of the individual amino acids was divided by the average content in the entire human proteome.

Table S1. Parameters for the fits of the Mössbauer spectra of human and yeast Mak16.

Sample	Temp. (K)	δ (mm/s)[a]	ΔE_Q (mm/s) [a]	Γ (mm/s) [a]	η	Occup. (%) [a]
$[4Fe\text{-}4S]^{2+}$ as isolated Mak16 *Homo sapiens*	77	0.42	0.99	0.31		50
		0.44	1.31	0.31		50
$[4Fe\text{-}4S]^{1+}$ dithionite treated Mak16 *Homo sapiens* (75 % red.)[b]	77	0.54	0.90	0.38		37.5
		0.61	1.52	0.51		37.5
$[4Fe\text{-}4S]^{2+}$ as isolated Mak16/Rpf1-Δ58 *Saccharomyces cerevisiae*[c]	77	0.43	1.03	0.26		50
		0.45	1.30	0.27		50
$[3Fe\text{-}4S]^{1+}$ as isolated Mak16/ Rpf1-Δ58 *Saccharomyces cerevisiae*[c]	77	0.28	0.55	0.30		15
Fe^{2+} 5 mM H_2O_2 treated Mak16/ Rpf1-Δ58 *Saccharomyces cerevisiae*[c]	77	0.55	2.90	0.40		5
$[4Fe\text{-}4S]^{2+}$ as isolated Mak16/Rpf1-Δ58 *Saccharomyces cerevisiae*	4	0.42	0.99	0.28	0	50
		0.44	1.29	0.29	0	50
$[4Fe\text{-}4S]^{1+}$ dithionite treated Mak16/ Rpf1-Δ58 *Saccharomyces cerevisiae* (100% red.)	77	0.53	0.96	0.36		50
		0.58	1.33	0.36		50
$[4Fe\text{-}4S]^{1+}$ dithionite treated Mak16/ Rpf1-Δ58 *Saccharomyces cerevisiae* (100% red.)[d]	4	0.52	1.05	0.52	0.5	50
		0.58	1.27	0.52	0.5	50

[a] Experimental errors for the Mössbauer parameters are ± 0.02 mm/s. Occupancies have an accuracy of ± 3 %.

[b] Parameters for the oxidized form were kept fixed and were taken from the fit for the as isolated state (but with 12.5 and 12.5 % occupancy).

[c] The simulations of the two quadrupole doublets of the $[4Fe\text{-}4S]^{2+}$ cluster were based on the more concentrated non-treated sample in Fig. S15 (top left). However, the latter sample had a clear $[3Fe\text{-}4S]^{1+}$ component, contrary to the sample shown in Fig. 2F, which had < 5% of this component. The parameters for the $[3Fe\text{-}4S]^{1+}$ component were determined by fitting the Mössbauer difference spectrum of 5 mM H_2O_2 treated minus non treated. Just detectable ferrous ions were simulated with the parameters in this table.

[d] The spectra at 4 K were too noisy to determine the $A/g_N\beta_N$ tensors from the experiment. Therefore the values for the *Bacillus stearothermophilus* ferredoxin were used (12). Parameters for the $A/g_N\beta_N$ tensor were -23, -24 and -<u>19</u> T for the $Fe^{2.5+}$-$Fe^{2.5+}$ doublet, and <u>20</u>, 10 and <u>10</u> T for the Fe^{2+}-Fe^{2+} doublet. The underlined values were slightly adapted.

Table S2. Mössbauer parameters for $[4Fe\text{-}4S]^{2+}$ containing proteins.

Protein	Organism	Temp. (K)	δ (mm/s) $Fe^{2.5+}$	ΔE_Q (mm/s) $Fe^{2.5+}$	Occup. (%)	Ref.
HiPIP	*Allochromatium vinosum*	77	0.42	1.12	100	(13)
HiPIP	*Allochromatium vinosum*	77	0.42	1.13	100	(14)
Ferredoxin	*Bacillus stearothermophilus*	77	0.42	0.98	100	(15)
Ferredoxin[a]	*Bacillus stearothermophilus*	77	0.43	1.36	25[a]	(12)
			0.43	1.07	25[a]	
			0.43	0.86	25[a]	
			0.42	0.57	25[a]	
GPAT	*Bacillus subtilis*	100	0.43	1.17	100	(16)
Nitrogenase Fe protein	*Azotobacter vinelandii*	80	0.43	1.14	75	(17)
			0.425	0.77	25	
Photosystem I Fx	*Synechococcus sp*. PCC 6301	80	0.43	1.06	100	(18)
APS reductase[a]	*Desulfovibrio gigas*	100	0.42	1.28	25[a]	(19)
			0.43	1.11	25[a]	
			0.43	0.88	25[a]	
			0.41	0.55	25[a]	
Enoate reductase	*Clostridium tyrobutyricum*	130	0.43	1.315	100	(20)
Iron-sulfur flavoprotein	*Methanosarcina thermophila*	100	0.45	1.12	100	(21)
HypD	*Escherichia coli*	77	0.43	0.95	100	(22)
PsaC ferredoxin	*Synechococcus sp*. PCC 7002	80	0.44	1.07	100	(23)
ABCE1	*Sulfolobus solfataricus*	77	0.43	1.32	50	(24)
			0.42	0.86	50	
NreB	*Staphylococcus aureus*	80	0.44	1.16	100	(25)
Benzoyl-coenzyme A epoxidase	*Azoarcus evansii*	80	0.44	1.10	100	(26)
Cfd1/Nbp35	*Saccharomyces cerevisiae*	80	0.43	1.18	100	(27)
Benzylsuccinate synthase	*Thauera aromatica*	80	0.43	1.09	100	(28)
PhrB	*Agrobacterium tumefaciens*	80	0.42	1.26	100	(29)
Dre2	*Saccharomyces*	80	0.43	0.99	50	(30)
	cerevisiae		0.43	1.29	50	
Mak16	*Homo sapiens*	77	0.42	0.99	50	
			0.44	1.31	50	
Mak16	*Saccharomyces*	77	0.43	1.03	50	
	cerevisiae		0.45	1.30	50	

[a] The Mössbauer spectra were fitted with four quadrupole doublets, though theory clearly defines two Fe^{2+}-Fe^{3+} ($Fe^{2.5+}$) pairs, which should yield two doublets. For **Fig. 2C** in the main text the average of the isomer shifts and quadrupole splittings of the two doublets with the lowest and highest quadrupole splittings was used.

Table S3. EPR simulation parameters for Fig. 2D and S7B.

Protein	Weight	$g_{av.}$	g_z	g_y	g_x	w_{zz}	w_{yy}	w_{xx}	w_{yz}	w_{xz}	w_{xy}
Human	0.50	1.933	2.001	1.918	1.880	0.020	0.010	0.045	0.000	0.000	0.000
Mak16	0.30	1.931	2.010	1.905	1.878	0.009	0.010	0.017	0.000	0.000	0.000
(reduced)	0.20	1.952	2.055	1.910	1.890	0.025	0.010	0.032	0.000	0.000	0.000
Yeast Mak16 (oxidized)	1.00	2.001	2.026	2.008	1.968	0.006	0.010	0.027	0.000	0.004	0.010
Yeast Mak16	0.69	1.936	2.015	1.909	1.885	0.013	0.013	0.033	0.000	0.000	0.005
(reduced)	0.29	1.947	2.071	1.890	1.880	0.016	0.015	0.030	0.000	0.000	0.000
S = 3/2	0.02	3.40	4.50	3.33	1.90	0.5	1.0	2.8	0.000	0.000	0.000
S = 5/2	0.0002	4.28	4.28	4.28	4.28	0.07	0.07	0.07	0.000	0.000	0.000

Table S4. Mössbauer parameters for $[4Fe\text{-}4S]^{1+}$ containing proteins.

Protein	Organism	Temp. (K)	δ (mm/s) $Fe^{2.5+}$	ΔE_Q (mm/s) $Fe^{2.5+}$	δ (mm/s) Fe^{2+}	ΔE_Q (mm/s) Fe^{2+}	Ref.
Ferredoxin	*Bacillus stearothermophilus*	77	0.50	1.18	0.60	1.82	(15)
Ferredoxin	*Bacillus stearothermophilus*	77	0.49	1.20	0.59	1.84	(12)
Ferredoxin	*Desulfovibrio gigas*	90	0.51	1.07	0.60	1.67	(31)
Nitrogenase Fe protein HMPA	*Azotobacter vinelandii*	50	0.53	0.94	0.58	1.51	(17)
Nitrogenase Fe protein ethyleneglycol	*Azotobacter vinelandii*	50	0.53	0.98	0.59	1.60	(17)
APS reductase cluster I	*Desulfovibrio gigas*	70	0.51	1.26	0.60	2.00	(19)
APS reductase cluster II	*Desulfovibrio gigas*	100	0.50	0.85	0.57	1.35	(19)
Enoate reductase	*Clostridium tyrobutyricum*	100	0.50	1.22	0.61	2.32	(20)
Iron-sulfur flavoprotein	*Methanosarcina thermophila*	100	0.50	0.90	0.57	1.68	(21)
Mak16	*Homo sapiens*	77	0.54	0.90	0.61	1.52	
Mak16	*Saccharomyces cerevisiae*	77	0.53	0.96	0.58	1.33	
Ferredoxin	*Clostridium pasteurianum*	77	0.57	1.25	0.57[a]	1.25[a]	(32)
GPAT	*Bacillus subtilis*	100	0.56	1.05	0.56[a]	1.05[a]	(16)
Nitrogenase Fe protein urea	*Azotobacter vinelandii*	50	0.54	0.80	0.54[a]	1.20[a,b]	(17)
Photosystem I Fx	*Synechococcus sp.* 6301	80	0.55	1.21	0.55[a]	1.21[a]	(18)
Activator HgdC	*Acidaminococcus fermentans*	80	0.53	0.95	0.53[a]	0.95[a]	(33)
PsaC ferredoxin	*Synechococcus sp.* PCC 7002	80	0.53	1.13	0.53[a]	1.13[a]	(23)

[a] The Mössbauer spectra were fitted with a single quadrupole doublet with parameters, which appear to be in between Mössbauer parameters for the Fe^{2+}-Fe^{3+} ($Fe^{2.5+}$) and Fe^{2+}-Fe^{2+} pairs.
[b] As in [a], but with two doublets with different linewidths.

Table S5. Primers for the construction of yeast strains.

Name	Sequence (5‘ → 3‘)	Strain
**Mak16_S1**	GTG ATT GGT ATA GAG GCA GAT ATT GCA TTG CTT AGT TCT TTC TTT TGA TGC GTA CGC TGC AGG TCG AC	Gal-*MAK16*
**Mak16_S4**	CTA TGA GAG CAG AAA CTT TGA TTA ATC ACT TGC CAA ACA ATT TCG TCG GAC ATC GAT GAA TTC TCT GTC G	
**Mak16_For**	GAT TCC CTC GCT AGA CAG G	
**Mak16_Rev**	GTG CTA CAC CAA CGT AGT GTC	
**Gal1_prom_Nbp35**	CGC ATT CAT TAA TGG TGT AAT TAG TAA TGC GTA GAA ATA TTT TGA TCG ATG AAT TCG AGC TC	Gal-*NBP35*
**Gal1_orf_Nbp35**	CTG CTG GTA GCA CTT CGT CGT TTA CAT GTG GTA GTA TCT CAG TCA TCG AAT TCC TTG AAT TTT C	
**Check_Nbp35_For**	CAC AAT TCT CTC AGT TCT ATG C	
**Check_Nbp35_Rev**	AAG TGC CCA ACT TAG CAT TGC	
**GalL_prom_Cia2**	AGT CGA ATA TAA AAT CTT GCT ACT GCA TTA TTC TAC GAT TTC CTG TTA TCG TAC GCT GCA GGT CGA C	Gal-*CIA2*
**GalL_orf_Cia2**	AGT TGG TTC TCC TCT AAA ATG TCG GGA TTT TCA TTC AAA AAC TCA GAC ATC GAT GAA TTC TCT GTC G	
**Check_Cia2_For**	CGC AAT GCT TGG GTG TAC GG	
**Check_Cia2_Rev**	GAC TTA ACG GGT GCT CAG GG	
**Cfd1_prom_control**	CAC ATC GAT AAG AGT GAA ACA TAT CC	Gal-*MAK16/*<br>Gal-*CFD1*
**Cfd1_354-Rev**	CAT AGA AGT CTT CTT AGG ACC C	
**GalCia1_Nterm**	CAT ATC TTT TCC TTG TAA AGT TTC AAA GAC TTA ATC AGA TTG ATA GAC GCC ATC GAA TTC CTT GAA TTT TC	Gal-*MAK16/*<br>Gal-*CIA1*
**PromCia1_control**	GCA CTT CTA- TCT TGT TTT GTG TTG C	
**GalL_Rpf1_S1**	CGA TTG AAC ACCT AA GGA AGA GGT AAA GCA GGC TTA ATA TAT TGA AAT ACG TAC GCT GCA GGT CGA C	Gal-*MAK16/*<br>Gal-*RPF1*
**GalL_Rpf1_S4**	TCT TGC CTC TTT AGC TTG TTT GTG ATG TTT ATC TCA TTA CCG AGA GCC ATC GAT GAA TTC TCT GTC G	
**Rpf1_331-Rev**	CAA ATT CAT AAG CGC TCT TCT TAG CG	
**Rpf1_–169-For**	TCA GTG CAT TTT TCA TTT TCG CGT TCG	

Table S6. Sequencing primers.

Name	Sequence (5‘ → 3‘)
**Seq1_Mak16**	GAT TCC CTC GCT AGA CAG G
**Seq2_Mak16**	GTC CGA CGA AAT TGT TTG GC
**Seq3_Mak16_Rev**	GGT TGT TAT TAT GGT ACA GTG CG
**Cia1_Seq1**	GAA GCG CTT CGG AAT TCA G
**Cia1_Seq2**	CTC ACA CTT CGC TGT TAG C
**Cia1_Seq3_Rev**	GCG TTA TAG TGA TAC AAA GG
**Rpf1–For**	CCT TCG TGT AAT ACA GGG TCG
**Rpf1–Rev**	GGG CGT GAA TGT AAG CGT GAC
**Cfd1–For**	AGT AAC TAC CCC ACA GAG TGT TGC
**pRS_4xx_SacI**	GGA TAA CAA TTT CAC ACA GG
**T7_forward**	TAA TAC GAC TCA CTA TAG G
**T7_reverse**	TGC TAG TTA TTG CTC AGC GG
**Cyc–50_Rev**	GGA CCT AGA CTT CAG GTT G
**His_seq310**	CTC ACA GAC GCG TTG AAT TG

Table S7. *Saccharomyces cerevisiae* strains.

Strain	Genotype	Background strain	Template	Ref.
**W303-1A**	MATa *leu2–3, 112 his3–11, 15 trp1–1, can1–100, ade2–1, ura 3–1*	–	–	(34)
**Gal-*NFS1***	*W303*–1A *pNFS1*::*HIS3*–p*GAL1–10*	–	–	(35)
**Gal-*NAR1***	*W303*–1A *pNAR1*::*HIS3*–p*GAL1–10*	–	–	(36)
**Gal-*CIA2***	*W303*–1A *pCIA2*:: *natNT2–GALL*	W303-1A	pYM_N27 (*Amp^R*, *natNT2)*	this work
**Gal-*CIA1***	*W303*–1A *pCIA1*::*HIS3*–p*GAL1–10*	–	–	(37)
**Gal-*CFD1***	*W303*–1A *pCFD1*::*HIS3*–*pGAL1–10*	–	_	(37)
**Gal-*MAK16***	*W303*–1A *pMAK16*:: *natNT2–GALL*	W303-1A	pYM_N27 (*Amp^R*, *natNT2)*	this work
**Gal-*MAK16*/Gal-*NBP35***	*W303*–1A *pMAK16*:: *natNT2–GALL, pNBP35*::*HIS3–pGAL1–10*	Gal-*MAK16*	pFA6a–HisMX6-Gal1-10 (*Amp^R*, *HIS3MX6)*	this work
**Gal-*MAK16*/Gal-*CIA1***	*W303*–1A *pMAK16*:: *natNT2*–p*GALL, pCIA1*::*HIS3*–p*GAL1–10*	Gal-*CIA1*	pYM_N27 (*Amp^R*, *natNT2)*	this work
**Gal-*MAK16*/Gal-*CFD1***	*W303*–1A *pMAK16*:: *natNT2–pGALL, pCFD1*::*HIS3–pGAL1–10*	Gal-*CFD1*	pYM_N27 (*Amp^R*, *natNT2)*	this work
**Gal-*MAK16*/Gal-*RPF1***	*W303*–1A *pMAK16*:: *natNT2–pGALL, pRPF1*::*HIS3–pGAL1–10*	Gal-*MAK16*	pYM_N27 with *natNT2* replaced by *HisMX6* (*Amp^R*, *HIS3MX6)*	this work

Table S8. *Escherichia coli* strains.

Strain	Genotype
**NEB® 5-alpha (DH5α)**	*fhuA2Δ(argF–lacZ)U169 phoA glnV44 Φ80Δ(lacZ)M15 gyrA96 recA1 relA1*
**NEB® 10-beta**	*Δ(ara–leu)7697 araD139 fhuA ΔlacX74 galK16 galE15 e14–ϕ80dlacZΔM15 recA1 endA1 nupG rpsL (StrR) rph spoT1 Δ(mrr–hsdRMS–mcrBC)*
**NEB® 5-alpha F'I^q**	F´ *proA$^+$B$^+$ lacI^q* ∆*(lacZ)M15 zzf*::*Tn10* (Tet^R) */ fhuA2*∆*(argF–lacZ)U169 phoA glnV44 Φ80Δ(lacZ)M15 gyrA96 recA1 relA1*
**T7 Express *lysY/I^q***	MiniF *lysY lacI^q*(Cam^R) */ fhuA2 lacZ*::*T7 gene1 [lon] ompT gal sulA11 R(mcr–73*::*miniTn10*–Tet^S*)2 [dcm] R(zgb–210*::*Tn10*–Tet^S*) endA1 Δ(mcrC–mrr) 114*::*IS10*

Table S9. Cloning primers.

Constructed plasmid	Primers	Sequence (5' → 3')	Starting plasmid, template
**416-NP$_{MAK16}$ (*MAK16* nat. promoter yeast)**	NP$_{MAK16}$_SacI_For	GTA **GAG CTC** TTA GAA GAT AAA GTA GTG AAT TAC	SacI/XbaI cut 416-MET25, yeast genomic DNA
	NP$_{MAK16}$_XbaI_Rev	GTA **TCT AGA** ATC CTC GTT GTA TTC TAA CCG TTG	
**416-NP-Mak16 (yeast)**	NP$_{MAK16}$_SacI_For	GTA **GAG CTC** TTA GAA GAT AAA GTA GTG AAT TAC	SacI/XhoI cut 416-MET25, yeast genomic DNA
	Mak16_XhoI_Rev	ATG GTA **CTC GAG** TTA TTG TGC CAC TTC TTG CTC	
**416-*MET25*-HA-Mak16 (yeast)**	Mak16_XbaI_For	ATG GTA **TCT AGA** ATG TCC GAC GAA ATT GTT TGG	SpeI/XhoI cut 416-MET25-HA-Pol3CTD (38), yeast genomic DNA
	Mak16_XhoI_Rev	ATG GTA **CTC GAG** TTA TTG TGC CAC TTC TTG CTC	
**416-NP-Mak16 (*E. cuniculi*)**	Mak16Ecun_BamHI	TGG TA**G GAT CC**G ATG TCT GAC GAG AGC TTG TGG	BamHI/SalI cut 416-NP$_{MAK16}$, *E. cuniculi* genomic DNA
	Mak16Ecun_SalI	GGT A**GT CGA C**TC ACC ACT TCA TTG CTA CCT TCT	
**416-NP-Mak16 (*H. sapiens*)**	Mak16Hsap_BamHI	TGG TA**G GAT CC**C ATG CAG TCG GAT GAT GTT ATC	BamHI/SalI cut 416-NP$_{MAK16}$, human cDNA (clone MGC:57551)
	Mak16Hsap_SalI	TGG TA**G TCG AC**T CAC GTG GTT TTG GCT TTG GCC	
**416-NP-Mak16 (*T. brucei*)**	Mak16Tbru_BamHI	TGG TA**G GAT CC**G ATG AAC CAT GAT GAT GCG ATG	BamHI/SalI cut 416-NP$_{MAK16}$, *T. brucei* genomic DNA
	Mak16Tbru_SalI	GGT A**GT CGA C**TC ACC AAT CCA AAT CAG CCG TGC	
**416-*MET25*-HA-Mak16 (*E. cuniculi*)**	Mak16Ecun_SpeI	GGTA**ACTAGT**ATGTCTGACGAGAGCTTGTGGAG	SpeI/SalI cut 416-MET25-HA-Rev3CTD (38), 416-NP-Mak16 *(E. cuniculi)*
	Mak16Ecun_SalI	GGTA**GTCGAC**TCACCACTTCATTGCTACCTTCT	
**416-*MET25*-HA-Mak16 (*H. sapiens*)**	pRSxxx-SacI	GGA TAA CAA TTT CAC ACA GG (64 bp at the 5' of SacI in 416)	SacI/SpeI cut 416-NP-Mak16 (*H. sapiens*), 416-MET25-HA-Pol3CTD (38)
	3HA_SpeI_rev	GGT A**AC TAG T**TC TCT AGT ATT CTC TGT CGG ACC	
**416-*MET25*-HA-Mak16 (*T. brucei*)**	pRSxxx-SacI	GGA TAA CAA TTT CAC ACA GG (64 bp at the 5' of SacI in 416)	SacI/SpeI cut 416-NP-Mak16 (*T. brucei*), 416-MET25-HA-Pol3CTD (38)
	3HA_SpeI_rev	GGT A**AC TAG T**TC TCT AGT ATT CTC TGT CGG ACC	
**pETDuet-1-His$_6$Mak16 (*H. sapiens*)**	Mak16_BamHI_Hsfor	GCA CTG **GGA TCC** CAT GCA GTC GGA TGA TGT TAT CTG G	BamHI/SalI cut MCS1 of pETDuet-1, 416-NP-Mak16 (*H. sapiens*)
	Mak16_SalI_Hsrev	GGT A**GT CGA C**TC ACG TGG TTT TGG CTT TGG CCA CGG	
**pETDuet-1-His$_6$Mak16 (yeast)**	Mak16_BamHI_For	TGG TA**G GAT CC**T ATG TCC GAC GAA ATT GTT TGG	BamHI/SalI cut MCS1 of pETDuet-1, yeast genomic DNA
	Mak16_SalI_Rev	GGT A**GT CGA C**TT ATT GTG CCA CTT CTT GCT CAG	

**pETDuet-1-Rpf1 (MCS2)**	**NdeI_Rpf1_Sc_For(2)**	**GCA CTG** CAT ATG **GCT CTC GGT AAT GAG ATA AAC ATC**	**NdeI/XhoI cut MCS2 of pETDuet-1, yeast genomic DNA**
	XhoI_Rpf1_Rev	GCA CTG **CTC GAG** GTT TAC GAA TGG AAC ATA AAT AAA AC	
**pETDuet-1-His$_6$Mak16/Rpf1**	Mak16_BamHI_For	GCA CTG **GGA TCC** GAT GTC CGA CGA AAT TGT TTG GCA AGT G	BamHI/PstI cut MCS1 of pETDuet-1-Rpf1 (MCS2), yeast genomic DNA
	Mak16_PstI_Rev	GCA CTG **CTG CAG** TTA TTG TGC CAC TTC TTG CTC AGC	
**pETDuet-1-His$_6$Mak16/Rpf1-Δ58**	NdeI_Rpf1_Q58M_For	GCA CTG **CAT ATG** ACG ATA GAG AAC ACT AGA GTG TAC G	NdeI/XhoI cut pETDuet-1-His$_6$Mak16/Rpf1, yeast genomic DNA
	XhoI_Rpf1_Rev	GCA CTG **CTC GAG** GTT TAC GAA TGG AAC ATA AAT AAA AC	
**414-NP-Cia1**	Cia1_NP_SacI_For	ATG GTA **GAG CTC** CCG CCT GCT TAT TCG ACA TC	SacI/SalI cut 414-MET25, yeast genomic DNA
	Cia1_NP_SalI_Rev	ATG GTA **GTC GAC** CTA CGC TGC TTT TTC TAG AGA C	
**414-*MET25*-Myc-Rpf1**	Rpf1_SpeI_For	GCT CGT **ACT AGT** ATG GCT CTC GGT AAT GAG ATA AAC	SpeI/EcoRI cut 414-MET25-Myc-Cfd1 (27), yeast genomic DNA
	Rpf1_EcoRI_Rev	GCT CGT **GAA TTC** CGA ATG GAA CAC TAT AAA TAA AAC	

Table S10. Mutagenesis primers.

Constructed plasmid	Primers	Sequence (5‘ → 3‘)	Mutagenized plasmid
**416-NP-Mak16 (yeast) C15A**	Mak16_C15A_for<br>Mak16_C15A_rev	TCAAAGCTTCGCCTCTCATAGAATTAAGGCACC<br>CTATGAGAGGCGAAGCTTTGATTAATCACTTGC	416-NP-Mak16 wildtype (yeast)
**416-NP-Mak16 (yeast) C28A**	Mak16_C28A_for<br>Mak16_C28A_rev	GTCAAAATTTTGCCAGAAATGAGTATAACGTCA<br>TCATTTCTGGCAAAATTTTGACCATTAGGTGCC	416-NP-Mak16 wildtype (yeast)
**416-NP-Mak16 (yeast) C38A**	Mak16_C38A_for<br>Mak16_C38A_rev	CGTCACTGGGCTAGCTACAAGGCAATCATGCCC<br>TTGTAGCTAGCCCAGTGACGTTATACTCATTTC	416-NP-Mak16 wildtype (yeast)
**416-NP-Mak16 (yeast) C15A/C38A**	Mak16_C38A_for<br>Mak16_C38A_rev	CGTCACTGGGCTAGCTACAAGGCAATCATGCCC<br>TTGTAGCTAGCCCAGTGACGTTATACTCATTTC	416-NP-Mak16 C15A (yeast)
**416-NP-Mak16 (yeast) C43A**	Mak16_C43A_for<br>Mak16_C43A_rev	ATCAGCCCCGCTAGCCAACTCCAAGTATGCAAC<br>TTGGCTAGCGGGGCTGATTGCCTTGTACACAAC	416-NP-Mak16 wildtype (yeast)
**416-NP-Mak16 (yeast) C55A**	Mak16_C55A_for<br>Mak16_C55A_rev	ACAGTGAAGGCTGACAATGGGAAACTGTACTTG<br>CCATTGTCAGCCTTCACTGTTGCATACTTGGAG	416-NP-Mak16 wildtype (yeast)
**416-NP-Mak16 (yeast) C108A**	Mak16_C108A_for<br>Mak16_C108A_rev	CGTCATAAGGCTAAACAGAGATTTACAAAATTG<br>TCTGTTTAGCCTTATGACGGAAAAACTTGCTCC	416-NP-Mak16 wildtype (yeast)
**416-*MET25*-HA-Mak16 (yeast) C15A**	Mak16_C15A_for<br>Mak16_C15A_rev	TCAAAGCTTCGCCTCTCATAGAATTAAGGCACC<br>CTATGAGAGGCGAAGCTTTGATTAATCACTTGC	416-*MET25*-HA-Mak16 wildtype (yeast)
**416-*MET25*-HA-Mak16 (yeast) C28A**	Mak16_C28A_for<br>Mak16_C28A_rev	GTCAAAATTTTGCCAGAAATGAGTATAACGTCA<br>TCATTTCTGGCAAAATTTTGACCATTAGGTGCC	416-*MET25*-HA-Mak16 wildtype (yeast)
**416-*MET25*-HA-Mak16 (yeast) C38A**	Mak16_C38A_for<br>Mak16_C38A_rev	CGTCACTGGGCTAGCTACAAGGCAATCATGCCC<br>TTGTAGCTAGCCCAGTGACGTTATACTCATTTC	416-*MET25*-HA-Mak16 wildtype (yeast)
**416-*MET25*-HA-Mak16 (yeast) C15A/C38A**	Mak16_C38A_for<br>Mak16_C38A_rev	TCAAAGCTTCGCCTCTCATAGAATTAAGGCACC<br>CTATGAGAGGCGAAGCTTTGATTAATCACTTGC	416-*MET25*-HA-Mak16 C15A (yeast)
**416-*MET25*-HA-Mak16 (yeast) C55A**	Mak16_C55A_for<br>Mak16_C55A_rev	ACAGTGAAGGCTGACAATGGGAAACTGTACTTG<br>CCATTGTCAGCCTTCACTGTTGCATACTTGGAG	416-*MET25*-HA-Mak16 wildtype (yeast)

Table S11. Plasmids for yeast.

Plasmid	Promoter	Source gene	Use	Ref.
**416-*MET25***	*MET25*	–	^{55}Fe incorporation, IP RNA isolation	(4)
**414-MET25**	*MET25*	–	Cloning, as control, IP	(4)
**416-NP (yeast *MAK16* promotor, NP)**	*MAK16*	*S. cerevisiae*	Cloning, RNA isolation growth complementation	this work
**416-NP-Mak16**	*MAK16*	*S. cerevisiae*	RNA isolation growth complementation	this work
**416-NP-Mak16$_{E.cun}$**	*MAK16*	*E. cuniculi*	growth complementation	this work
**416-NP-Mak16$_{H.sap}$**	*MAK16*	*H. sapiens*	growth complementation	this work
**416-NP-Mak16$_{T. bru}$**	*MAK16*	*T. brucei*	growth complementation	this work
**416-*MET25*-HA-Mak16**	*MET25*	*S. cerevisiae*	^{55}Fe incorporation, growth complementation	this work
**416-*MET25*-HA-Mak16$_{E.cun}$**	*MET25*	*E. cuniculi*	^{55}Fe incorporation, growth complementation	this work
**416-*MET25*-HA-Mak16$_{H.sap}$**	*MET25*	*H. sapiens*	^{55}Fe incorporation, growth complementation	this work
**416-*MET25*-HA-Mak16$_{T.bru}$**	*MET25*	*T. brucei*	^{55}Fe incorporation, growth complementation	this work
**416-NP-Mak16-C15A**	*MAK16*	*S. cerevisiae*	RNA isolation growth complementation	this work
**416-NP-Mak16-C28A**	*MAK16*	*S. cerevisiae*	RNA isolation growth complementation	this work
**416-NP-Mak16-C38A**	*MAK16*	*S. cerevisiae*	RNA isolation growth complementation	this work
**416-NP-Mak16-C15A/C38A**	*MAK16*	*S. cerevisiae*	RNA isolation growth complementation	this work
**416-NP-Mak16-C43A**	*MAK16*	*S. cerevisiae*	RNA isolation growth complementation	this work
**416-NP-Mak16-C55A**	*MAK16*	*S. cerevisiae*	growth complementation	this work
**416-NP-Mak16-C108A**	*MAK16*	*S. cerevisiae*	growth complementation	this work
**414-NP-Cia1**	*CIA1*	*S. cerevisiae*	growth complementation	this work
**414-*MET25*-Myc-Cfd1**	*MET25*	*S. cerevisiae*	growth complementation	(27)
**414-*MET25*-Myc-Nbp35**	*MET25*	*S. cerevisiae*	growth complementation	(27)
**414-*MET25*-Myc-Rpf1**	*RPF1*	*S. cerevisiae*	IP	this work
**416-*MET25*-HA-Mak16-C28A**	*MET25*	*S. cerevisiae*	IP	this work
**416-*MET25*-HA-Mak16-C38A**	*MET25*	*S. cerevisiae*	IP	this work
**416-*MET25*-HA-Mak16-C15A/C38A**	*MET25*	*S. cerevisiae*	IP	this work
**416-*MET2*5-HA-Mak16-C55A**	*MET25*	*S. cerevisiae*	IP	this work

Table S12. Plasmids for expression in *Escherichia coli*.

Name	Features	Ref.
**pETDuet-1-His$_6$Mak16_H. sap**	MCSI: His$_6$-Mak16 human	this work
**pETDuet-1-His$_6$Mak16/Rpf1 yeast**	MCSI: His$_6$-Mak16 yeast<br>MCSII: Rpf1 yeast full length	this work
**pETDuet-1-His$_6$Mak16/Rpf1-Δ58 yeast**	MCSI: His$_6$-Mak16 yeast<br>MCSII: Rpf1-Δ58 yeast	this work

Table S13. Antibodies.

Antibodies	Type	Dilution	Source
**Anti-HA (F-7) sc-7392**	monoclonal	1:1000 or 1:2000	Santa Cruz Biotechnology
**Anti-Myc (9E10) sc-40**	monoclonal	1:2000	Santa Cruz Biotechnology
**Anti-His (1-21315)**	monoclonal	1:1000	Invitrogen
**Anti-Nfs1**	polyclonal	1:1000	Rabbit (Prof. Lill, Marburg)
**Anti-Nar1**	polyclonal	1:1000	Rabbit (Prof. Lill, Marburg)
**Anti-Cia2**	polyclonal	1:1000	Rabbit (Prof. Lill, Marburg)
**Anti-Porin**	polyclonal	1:2000	Rabbit (Prof. Lill, Marburg)
**Goat anti-rabbit IgG-HRP**	monoclonal	1:5000	Santa Cruz Biotechnology
**Goat-anti mouse IgG-HRP**	monoclonal	1:10000	Santa Cruz Biotechnology

SI References

1. C. Janke, *et al*., A versatile toolbox for PCR-based tagging of yeast genes: new fluorescent proteins, more markers and promoter substitution cassettes. *Yeast* **21**, 947–962 (2004).
2. R. D. Gietz, R. A. Woods, Transformation of yeast by lithium acetate/single-stranded carrier DNA/polyethylene glycol method. *Methods Enzymol.* **350**, 87–96 (2002).
3. U. Mühlenhoff, N. Richhardt, M. Ristow, G. Kispal, R. Lill, The yeast frataxin homolog Yfh1p plays a specific role in the maturation of cellular Fe/S proteins. *Hum. Mol. Genet.* **11**, 2025–2036 (2002).
4. D. Mumberg, R. Müller, M. Funk, Yeast vectors for the controlled expression of heterologous proteins in different genetic backgrounds. *Gene* **156**, 119–122 (1995).
5. L. Zheng, U. Baumann, J.-L. Reymond, An efficient one-step site-directed and site-saturation mutagenesis protocol. *Nucleic Acids Res.* **32**, e115 (2004).
6. J. A. Gibson, M. Marshall, The counting efficiency for ^{55}Fe and other E.C. nuclides in liquid scintillator solutions. *Int. J. Appl. Radiat. Isot.* **23**, 321–328 (1972).
7. A. J. Pierik, R. B. Wolbert, P. H. Mutsaers, W. R. Hagen, C. Veeger, Purification and biochemical characterization of a putative [6Fe-6S] prismane-cluster-containing protein from *Desulfovibrio vulgaris* (Hildenborough). *Eur. J. Biochem.* **206**, 697–704 (1992).
8. W. R. Hagen, Very Low-Frequency Broadband Electron Paramagnetic Resonance Spectroscopy of Metalloproteins. *J. Phys. Chem. A* **125**, 3208–3218 (2021).
9. V. Schünemann, “From Small Molecules to Complex Systems: A Survey of Chemical and Biological Applications of the Mössbauer Effect” in *Y. Yoshida, G. Langouche (Eds.) 2021 – Modern Mössbauer Spectroscopy*, pp. 173–219.
10. H. P. Gunnlaugsson, Spreadsheet based analysis of Mössbauer spectra. *Hyperfine Interact.* **237**, 79 (2016).
11. F. Corpet, Multiple sequence alignment with hierarchical clustering. *Nucleic Acids Res.* **16**, 10881–10890 (1988).
12. P. Middleton, D. P. Dickson, C. E. Johnson, J. D. Rush, Interpretation of the Mössbauer spectra of the four-iron ferredoxin from *Bacillus stearothermophilus*. *Eur. J. Biochem.* **88**, 135–141 (1978).
13. M. C. Evans, D. O. Hall, C. E. Johnson, Hyperfine structure of ^{57}Fe iron in the Mössbauer spectrum of the high-potential iron protein from *Chromatium*. *Biochem. J.* **119**, 289–291 (1970).
14. D. P. Dickson, *et al*., Mössbauer effect in the high-potential iron-sulphur protein from *Chromatium*. Evidence for the state of the iron atoms. *Biochem. J.* **139**, 105–108 (1974).
15. R. N. Mullinger, *et al*., Physicochemical characterization of the four-iron-four-sulphide ferredoxin from *Bacillus stearothermophilus*. *Biochem. J.* **151**, 75–83 (1975).
16. S. J. Vollmer, R. L. Switzer, P. G. Debrunner, Oxidation-reduction properties of the iron-sulfur cluster in *Bacillus subtilis* glutamine phosphoribosylpyrophosphate amidotransferase. *J. Biol. Chem.* **258**, 14284–14293 (1983).
17. P. A. Lindahl, E. P. Day, T. A. Kent, W. H. Orme-Johnson, E. Münck, Mössbauer, EPR, and magnetization studies of the *Azotobacter vinelandii* Fe protein. *J. Biol. Chem.* **260**, 11160–11173 (1985).
18. V. Petrouleas, J. J. Brand, K. G. Parrett, J. H. Golbeck, A Mössbauer analysis of the low-potential iron-sulfur center in photosystem I: spectroscopic evidence that F_X is a 4Fe-4S cluster. *Biochemistry* **28**, 8980–8983 (1989).
19. J. Lampreia, *et al*., The active centers of adenylylsulfate reductase from *Desulfovibrio gigas*. Characterization and spectroscopic studies. *Eur. J. Biochem.* **188**, 653–664 (1990).
20. J. Caldeira, *et al*., EPR and Mössbauer spectroscopic studies on enoate reductase. *J. Biol. Chem.* **271**, 18743–18748 (1996).
21. D. F. Becker, U. Leartsakulpanich, K. K. Surerus, J. G. Ferry, S. W. Ragsdale, Electrochemical and spectroscopic properties of the iron-sulfur flavoprotein from *Methanosarcina thermophila*. *J. Biol. Chem.* **273**, 26462–26469 (1998).

22. M. Blokesch, *et al*., The complex between hydrogenase-maturation proteins HypC and HypD is an intermediate in the supply of cyanide to the active site iron of NiFe-hydrogenases. *J. Mol. Biol.* **344**, 155–167 (2004).

23. M. L. Antonkine, *et al.*, Chemical rescue of a site-modified ligand to a [4Fe-4S] cluster in PsaC, a bacterial-like dicluster ferredoxin bound to Photosystem I. *Biochim. Biophys. Acta* **1767**, 712–724 (2007).

24. D. Barthelme, *et al.*, Structural organization of essential iron-sulfur clusters in the evolutionarily highly conserved ATP-binding cassette protein ABCE1. *J. Biol. Chem.* **282**, 14598–14607 (2007).

25. M. Müllner, *et al*., A PAS domain with an oxygen labile $[4Fe\text{-}4S]^{2+}$ cluster in the oxygen sensor kinase NreB of *Staphylococcus carnosus*. *Biochemistry* **47**, 13921–13932 (2008).

26. L. J. Rather, E. Bill, W. Ismail, G. Fuchs, The reducing component BoxA of benzoyl-coenzyme A epoxidase from *Azoarcus evansii* is a [4Fe-4S] protein. *Biochim. Biophys. Acta* **1814**, 1609–1615 (2011).

27. D. J. Netz, *et al*., A bridging [4Fe-4S] cluster and nucleotide binding are essential for function of the Cfd1-Nbp35 complex as a scaffold in iron-sulfur protein maturation. *J. Biol. Chem.* **287**, 12365–12378 (2012).

28. M. Hilberg, *et al*., Identification of FeS clusters in the glycyl-radical enzyme benzylsuccinate synthase via EPR and Mössbauer spectroscopy. *J. Biol. Inorg. Chem.* **17**, 49–56 (2012).

29. T. O. Bauer, D. Graf, T. Lamparter, V. Schünemann, Characterization of the photolyase-like iron sulfur protein PhrB from *Agrobacterium tumefaciens* by Mössbauer spectroscopy. *Hyperfine Interact.* **226**, 445–449 (2014).

30. D. J. Netz, *et al*., The conserved protein Dre2 uses essential [2Fe-2S] and [4Fe-4S] clusters for its function in cytosolic iron-sulfur protein assembly. *Biochem. J.* **473**, 2073–2085 (2016).

31. J. J. Moura, *et al*., Interconversions of [3Fe-3S] and [4Fe-4S] clusters. Mössbauer and electron paramagnetic resonance studies of *Desulfovibrio gigas* ferredoxin II. *J. Biol. Chem.* **257**, 6259–6267 (1982).

32. C. L. Thompson, *et al*., Mössbauer effect in the eight-iron ferredoxin from *Clostridium pasteurianum*. Evidence for the state of the iron atoms. *Biochem. J.* **139**, 97–103 (1974).

33. M. Hans, W. Buckel, E. Bill, The iron-sulfur clusters in 2-hydroxyglutaryl-CoA dehydratase from *Acidaminococcus fermentans*. Biochemical and spectroscopic investigations. *Eur. J. Biochem.* **267**, 7082–7093 (2000).

34. B. J. Thomas, R. Rothstein, Elevated recombination rates in transcriptionally active DNA. *Cell* **56**, 619–630 (1989).

35. U. Mühlenhoff, *et al*., Functional characterization of the eukaryotic cysteine desulfurase Nfs1p from *Saccharomyces cerevisiae*. *J. Biol. Chem.* **279**, 36906–36915 (2004).

36. J. Balk, A. J. Pierik, D. J. A. Netz, U. Mühlenhoff, R. Lill, The hydrogenase-like Nar1p is essential for maturation of cytosolic and nuclear iron–sulphur proteins. *EMBO J.* **23**, 2105–2115 (2004).

37. J. Balk, D. J. Netz, M. Stümpfig, U. Mühlenhoff, R. Lill, The essential WD40 protein Cia1 is involved in a late step of cytosolic and nuclear iron-sulfur protein assembly. *Mol. Cell. Biol.* **25**, 10833–10841 (2005).

38. D. J. Netz, *et al*., Eukaryotic DNA polymerases require an iron-sulfur cluster for the formation of active complexes. *Nat. Chem. Biol.* **8**, 125–132 (2011).
